# Top-down and bottom-up effects modulate species co-existence in a context of top predator restoration

**DOI:** 10.1038/s41598-023-31105-w

**Published:** 2023-03-13

**Authors:** Tamara Burgos, Javier Salesa, Jose María Fedriani, Gema Escribano-Ávila, José Jiménez, Miha Krofel, Inmaculada Cancio, Javier Hernández-Hernández, Javier Rodríguez-Siles, Emilio Virgós

**Affiliations:** 1 Área de Biodiversidad y Conservación, Departamento de Biología, Geología, Física y Química Inorgánica, Rey Juan Carlos University, Madrid, Spain; 2 Centro de Investigaciones sobre Desertificación CIDE, CSIC-UVEG-GV, Carretera de Moncada a Náquera, km 4,5., 46113 Moncada, Valencia Spain; 3 Estación Biológica de Doñana (EBD – CSIC), Seville, Spain; 4 Biodiversity, Ecology and Evolution Department. Biological Science Faculty, Universidad Complutense de Madrid. Ciudad Universitaria, C/ José Antonio Novais 12, Madrid, Spain; 5 Instituto de Investigación en Recursos Cinegéticos (CSIC-UCLM-JCCM), 13071 Ciudad Real, Spain; 6 Asociación de Estudio y Conservación de Fauna Harmusch, C/San Antón 15, 1°, 13580 Almodóvar del Campo, Ciudad Real Spain; 7 Department for Forestry, Biotechnical Faculty, University of Ljubljana, Ljubljana, Slovenia; 8 Road Ecology Lab, Department of Biodiversity, Ecology and Evolution, Faculty of Biology, Complutense University of Madrid, Madrid, Spain

**Keywords:** Ecology, Behavioural ecology, Zoology, Animal behaviour

## Abstract

Mesopredators abundance is often limited by top-order predators and also by key food resources. However, the contribution of these bidirectional forces to structure carnivore community is still unclear. Here, we studied how the presence and absence of an apex predator which is currently recovering its former distribution range, the Iberian lynx (*Lynx pardinus*), determined the absolute abundance and fine-scale spatiotemporal avoidance mechanisms of two sympatric mesocarnivores (stone marten *Martes foina* and common genet *Genetta genetta*) with different dietary plasticity. We hypothesized that the lynx causes a mesopredator suppression and subordinate predators develop segregation strategies in respect to their trophic niche breadth. We placed 120 camera-traps in Southern Spain for 8 months in two consecutive years to estimate mesocarnivore abundances by using SCR Bayesian models, prey availability and assess spatio-temporal patterns. We found that the lynx reduced mesocarnivore abundance up to 10 times. Stone marten, a mesopredator with a broad food resources spectrum, showed a total spatial exclusion with the apex predator. Meanwhile, fine-scale avoidance mechanisms allowed the genet to persist in low density inside lynx territories, probably taking advantage of high availability of its preferred prey. Thus, the strength of these top-down and bottom-up effects was rather species-specific. Given the recent recovery of large carnivore populations worldwide, variation in suppression levels on different mesopredator species could modify ecosystem functions provided by the carnivore community in contrasting ways.

## Introduction

Apex predators play critical roles in ecosystem functioning, and their conservation is paramount for enhancing and maintaining global biodiversity^1^. They are at the top of trophic webs and can exert a top-down regulation on prey populations by direct killing^2,3^ and behaviourally-mediated trophic cascades due to behavioural responses to predation risk^4^. However, food availability can also trigger bottom-up effects on predators limiting their numbers^5^, especially for dietary or habitat specialists^6,7^. Therefore, considering the recent recovery and rewilding processes involving large carnivores across the world^8^, a better understanding of how these bidirectional forces between predators and prey structure trophic webs would help to improve our knowledge on food web dynamics and to adopt well-informed conservation strategies at the whole community level^9^.

Top-down regulation from apex predators can also affect carnivores in lower trophic levels through complex intra-guild interactions^10^. Large carnivores can reduce abundances of medium-size predators (i.e. mesopredators) by exploitative competition or inter-specific killing^11,12^. Therefore, extirpation or significant reduction of top predator numbers can lead to a mesopredator release^13,14^. Nevertheless, according to the coexistence theory^15^, the negative impacts of competitive interactions can be relaxed by several factors such as high availability of resources or adequate environmental conditions^16,17^. Thus, sympatric species can avoid intra-guild competition by performing adaptive behavioural mechanisms, specifically by spatial, temporal and/or dietary niche segregation^18–20^. These adaptations take special relevance in complex carnivore communities, where niche partitioning could involve positive cost–benefit balance to facilitate the coexistence among carnivores^21–23^.

As a consequence of asymmetric competition with larger predators, subordinate species (i.e. smaller predators) are often restricted to safer but less productive habitats^20,24,25^, at the periphery of the dominant predator ranges, especially when resources are limited^13^. Local and intensive foraging of subordinate carnivores persisting in safe but restricted areas could markedly decrease the local abundance of susceptible prey species^14^. In contrast, some prey populations can spread as a result of a reduced overall predation pressure in areas frequented by a dominant predator^26,27^. Therefore, the presence of an apex predator could displace mesopredators to lower-quality habitats but also encourage certain specialized mesopredators to persist in areas with high risk of intra-guild predation due to a balanced effect of high prey abundance^12^. Prey availability is especially relevant in a scenario of apex predator restoration where responses to competition by subordinate predators could be more complex than just fear or death^24,28^. Other studies have suggested that prey abundance and preferences can drive coexistence patterns as a bottom-up force structuring carnivore communities^19,29,30^. For instance, Monterroso et al.^17^ found that predator species with high overlapping dietary niches can coexist locally by prey switching when the subordinate species has a wide trophic niche breadth, accordingly with the optimal foraging theory^31^.

In Mediterranean ecosystems of southern Spain, the recent recovery of the apex predator, the Iberian lynx (*Lynx pardinus*), allowed us to investigate how intra-guild competition and prey availability structured the predator community. Here, we studied the top-down suppression and coexistence mechanisms among two mesopredators and an apex predator, the Iberian lynx, through a natural experiment. We studied areas with and without predation risk and measured top-down effects on the abundance and spatio-temporal behaviour of mesocarnivores by using spatial capture-recapture (SCR) and time-to-encounter methods, which was rarely used in previous similar research on top-down suppression^26^. We focused on two sympatric mesocarnivores with a high trophic niche overlap but different dietary plasticity: the common genet (*Genetta genetta*), specialized on small rodents and the stone marten (*Martes foina*), a more generalist and opportunistic carnivore^32^. Moreover, we assess how the availability of food resources modulates this trophic web through bottom-up effects. We hypothesize that the presence of the apex predator leads to a reduced mesopredator density in comparison with lynx absence scenarios. Furthermore, we expect mesopredators to show species-specific segregation or coexistence mechanisms with the apex predator and that prey availability and trophic plasticity to play an important role in facilitating coexistence. Specifically, we predict that the trophic-specialist, the common genet, should be able to develop spatio-temporal mechanisms to coexist with the apex predator due to the increase of preferred prey abundance (rodents) inside lynx home ranges. In contrast, we expect that for the mesopredator with a wider trophic-niche breadth, the stone marten, it should be easier to avoid lynx home ranges because is able to feed on a wider food spectrum.

## Results

### Overall patterns

**Figure 1:**
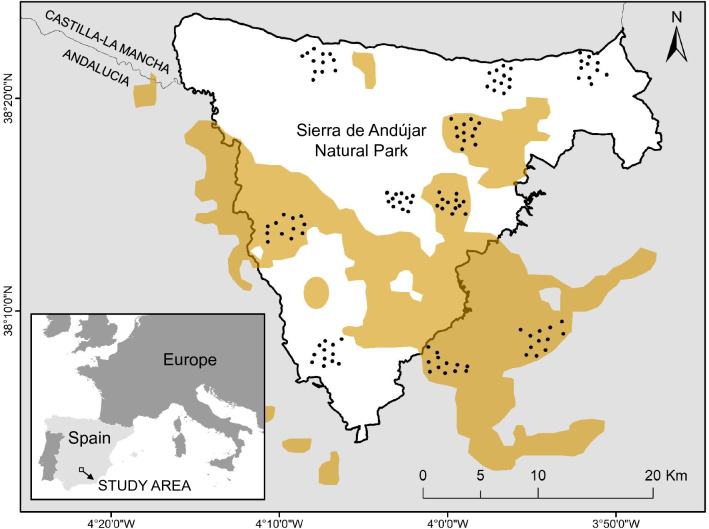
Map of study area showing the camera-trap sites (black dots) distributed in ten localities along the Sierra de Andújar NP and the Iberian lynx range (orange shadowed) adapted from the distribution map available in the IUCN Red List of Threatened Species^61^. Map was generated in ArcMap 10.6 https://www.esri.com/.

Camera-traps recorded 2.30 ± 0.56 (Mean ± SE) (n = 156), 13.79 ± 3.77 (n = 1546) and 21.59 ± 5.92 (n = 1708) independent captures per 100 cam-days of adult Iberian lynx, common genets, and stone martens, respectively. Only 22% of independent genet detections and 0.9% of marten detections were recorded at localities inside lynx range (Supplementary Appendix S3: Table S2). Genets and martens occupied, respectively, 41.6% and 5.8% of the camera-traps inside lynx territories and 80.8% and 87.5% outside (Supplementary Appendix S3: Table S2). We identified 5.0 ± 0.76 genets and 1.4 ± 0.24 stone martens per locality inside lynx territories, and 9.3 ± 1.16 genets and 9.3 ± 1.61 martens outside (Supplementary Appendix S3: Table S1). From the total of carnivore captures, 86.2%, 91.2% and 76.6% was recorded outside breeding season (March-June) for the lynx, genet and marten, respectively (Supplementary Appendix S3: Table S5). We recorded 3.5 ± 0.54 lynx adults per locality (Supplementary Appendix S3: Table S1) and we detected reproduction (kittens from the previous breeding season) in all ‘lynx presence’ localities. We found on average 55.8% ± 6.1% lynx occurrence and 2.9 ± 0.60 detections/100 cam-days (Supplementary Appendix S3: Table S2). We registered only four lynx detections (2.5% of the overall lynx detections in 2 years) in two localities considered as controls (Supplementary Appendix S3: Tables S1, S2), probably connected with sub-adult dispersal or occasional exploratory incursions of territorial males from territories in the vicinity (see Fig. 1). We did not register any lynx territorial couple or reproductive event within control localities.

Regarding prey species, detections by 100 cam-days were 80.62 ± 30.40 (n = 5445), 27.69 ± 6.29 (n = 2913) and 5.94 ± 1.38 (n = 416) for dormice, other rodents and rabbits respectively, of which the 98%, 73% and 99% were obtained inside lynx territories (see Supplementary Appendix S3: Table S3). Occurrence was 44.2% for rabbits, 57.5% for dormice and 80.8% for other rodents inside lynx ranges. In localities without lynx, the highest percentage of occupation was for other rodents (62.5%) but the garden dormouse and rabbit occupancy was very low (only 4.2% and 2.5%, respectively) (Supplementary Appendix S3: Table S3). We calculated that the 89.87% and 88.5% of the prey events occurred in lynx territories outside and inside breeding season, respectively (Supplementary Appendix S3: Table S5). During breeding season and considering together both predation risk scenarios, we detected 2.6 higher and 0.73 lower relative abundance than out of breeding for the garden dormouse and other rodents, respectively (Supplementary Appendix S3: Table S5).

### Mesocarnivore density estimates

**Figure 2:**
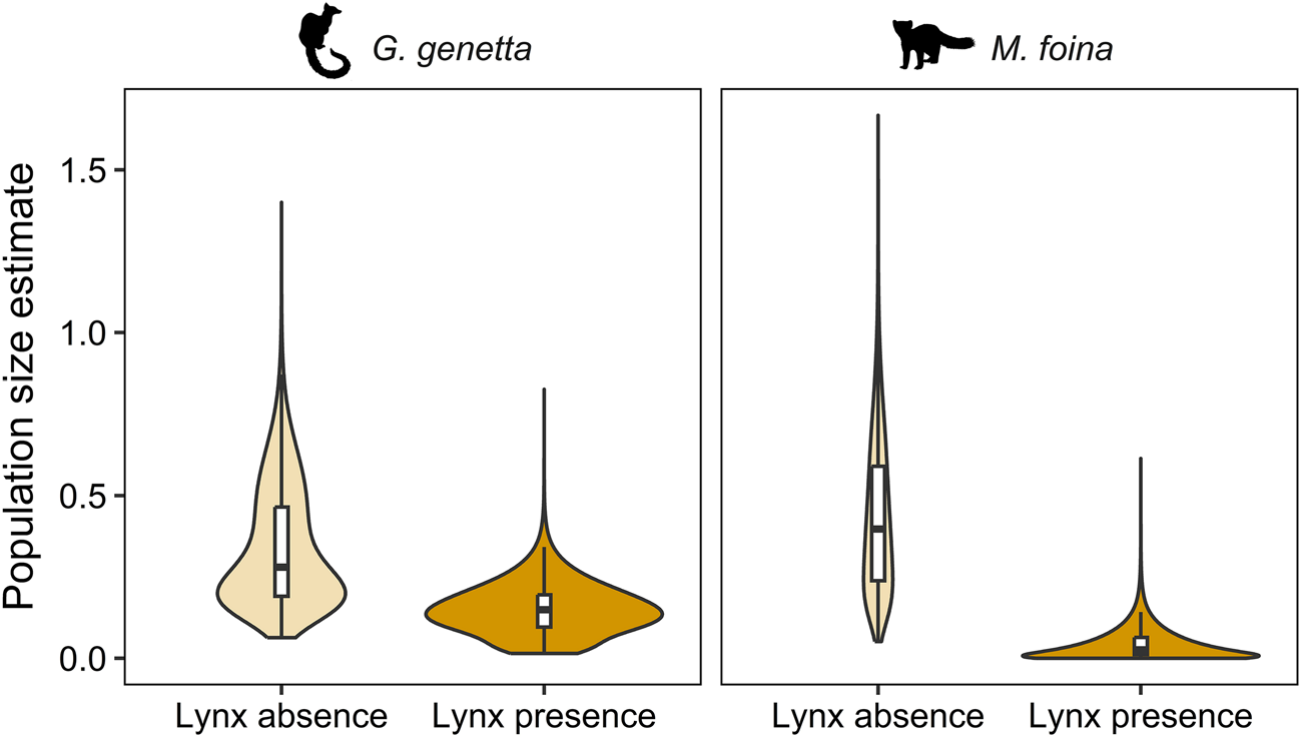
Estimated density (individuals per km^2^) for the common genet (*G. genetta*) and the stone marten (*M. foina*). The width of each density curve corresponds with the frequency of data in each abundance interval and boxplots show the median and data quartiles with upper and lower whisker limits.

The abundance of mesopredators (individuals/km^2^) was significantly higher outside lynx ranges (Table 1) and we observed stronger suppression on the stone marten density (Mean ± SE: 0.43 ± 0.07 vs. 0.04 ± 0.01; 10.75-fold decrease in lynx ranges) than on the common genet density (0.33 ± 0.05 vs. 0.15 ± 0.02; 2.20-fold decrease in lynx ranges) (Supplementary Appendix S2: Table S1, Fig. 2). At level of locality, the lowest stone marten densities always occurred in localities with lynx (Supplementary Appendix S2: Table S1). We did not find a significant effect from prey availability on carnivore density, even considering interactions among lynx presence/absence and prey species (Table 1).

**Table 1:** Coefficients with standard errors (SE), F or Likelihood ratio test statistics, significance values and marginal and conditional pseudo-R^2^ for the independent variables included in the generalized lineal mixed models (GLMM) for carnivore density, median time-to-encounter and encounter probability responses.

Response	Df	Fixed effects	Coeff. ± SE	F/LRT	P	R^2^_m_	R^2^_c_
Carnivore density	1	Intercept	− 1.24 ± 0.22	*–*	*–*	0.75	0.79
	1	L	− 0.76 ± 0.26	8.45	< 0.01**		
	1	CS	0.19 ± 0.17	1.20	> 0.05		
	1	PRAI	− 0.02 ± 0.43	0.001	> 0.05		
	1	L*CS	− 1.54 ± 0.22	50.94	< 0.0001***		
	1	L*PRAI	0.06 ± 0.46	0.02	> 0.05		
	2	PS:PRAI	*–*	0.10	> 0.05		
Median time-to-encounter	1	Intercept	2.32 ± 0.28	*–*	*–*	0.09	0.92
	1	GVR	0.25 ± 0.23	1.23	> 0.05		
	1	PVR	− 0.53 ± 0.31	2.96	> 0.05		
	1	PS	0.29 ± 0.19	2.37	> 0.05		
	1	PS:PVR	*–*	2.17	> 0.05		
Encounter probability	1	Intercept	− 23.48 ± 6.50	*–*	*–*	0.10	0.99
	1	OR	12.80 ± 4.79	13.49	< 0.001***		
	1	GD	33.16 ± 9.34	15.88	< 0.001***		

The variance of the random effect ‘locality’ was 0.01 ± 0.08 (σ ± SE) for carnivore density response. The variance of the random effect ‘cam’ was 0.59 ± 0.77 for median time-to-encounter response and 2630 ± 51.28 for the encounter probability.

*L lynx presence/absence, *CS* carnivore species, *PRAI* prey relative abundance index, *PS* prey species, *GVR* genet visitation rate, *PVR* prey visitation rate, *OR* other rodents presence/absence, *GD* garden dormouse presence/absence.

Significant values are in italic.

### Temporal overlap among lynx, mesocarnivores and prey

**Figure 3:**
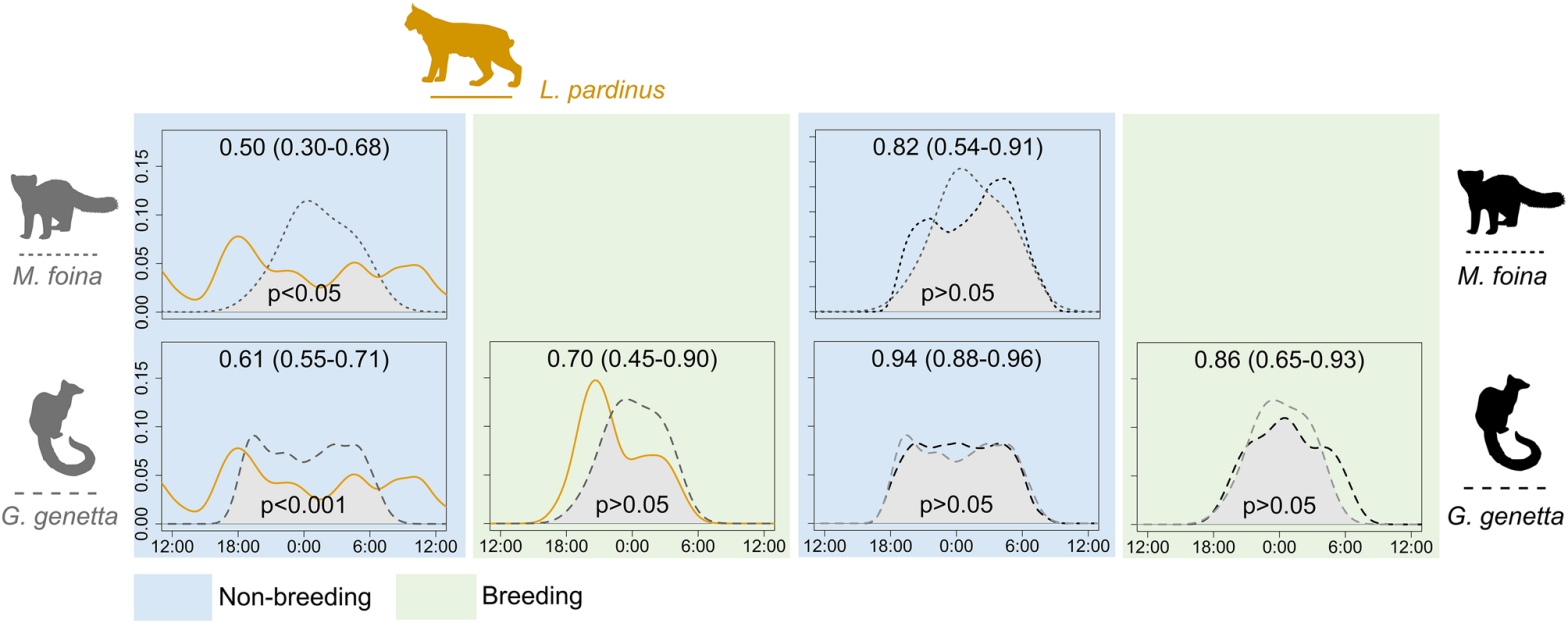
Temporal activity patterns of carnivores in localities with Iberian lynx presence (lynx silhouette) and localities without lynx (no lynx silhouette) outside the breeding season (blue boxes) and inside (green boxes). The Iberian lynx is represented by orange solid line, the stone marten by dotted line and the common genet by dashed line. Mesocarnivores co-existing with lynx are coloured in dark grey and mesocarnivores living outside lynx ranges in black. Empty plots refer to overlap unrealized combinations.

Lynx activity was mostly crepuscular with some of diurnal activity, but turned into more nocturnal during the breeding period (Fig. 3). Genet and marten showed a strictly nocturnal behaviour in all periods and predation risk scenarios (Fig. 3). Genets showed a clear peak of activity after sunset inside lynx ranges outside breeding season but their activity pattern was more uniform in localities without lynx, showing a later peak around midnight during the breeding season (Fig. 3). Martens showed a single activity peak around midnight when inhabiting Iberian lynx ranges and two peaks of activity in localities without lynx (Fig. 3). We were not able to calculate the activity patterns of stone marten inside lynx territories during breeding season because we did not get enough records due to its very low abundance in this scenario (Supplementary Appendix S3: Table S5). We found significant differences between lynx and genet and lynx and marten activity patterns outside breeding, with a low temporal overlap (Fig. 3). However, lynx-genet overlap during the breeding season was intermediate, showing non-significant differences in their activity patterns (Fig. 3). There was high overlap in genet and marten activity patterns comparing predation risk scenarios for both reproductive periods (Fig. 3).

**Figure 4:**
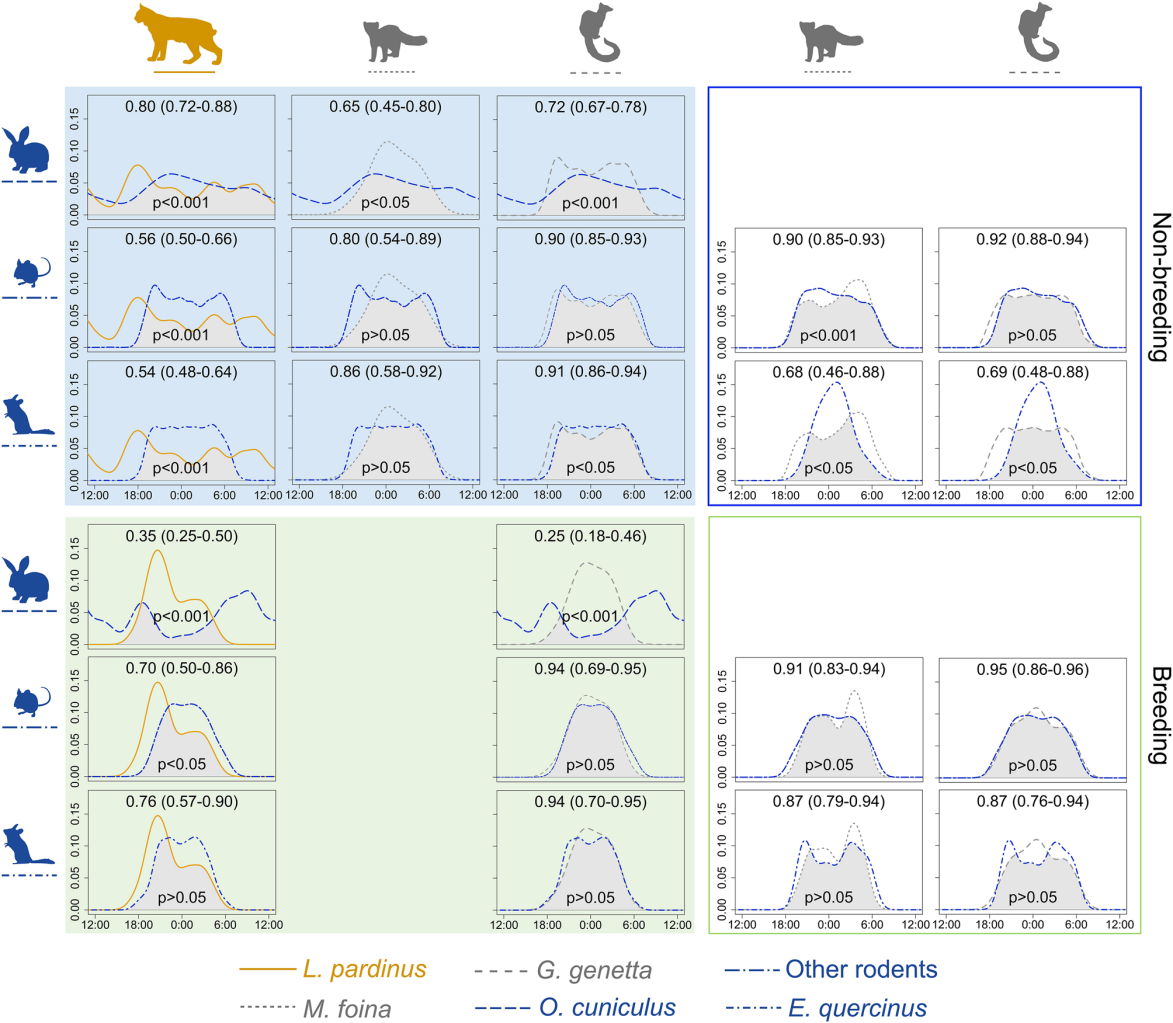
Temporal activity patterns of carnivores and prey species in localities with Iberian lynx presence (colour-filled boxes) and localities without lynx (non-colour filled boxes) outside breeding season (blue) and inside (green). Apex predator is represented by orange, mesocarnivores by grey and prey by blue. Iberian lynx (solid line), common genet (dashed line), stone marten (dotted line), European rabbit (long dash line), other rodents (long dot–dash line) and garden dormouse (dot–dash line). Empty plots refer to overlap unrealized combinations.

Rabbits showed a high activity overlap with the Iberian lynx outside breeding period and low overlap inside breeding season (Fig. 4). We found significant differences between rabbits and mesocarnivore activity patterns inside lynx ranges during both reproductive periods (Fig. 4). The activity pattern of rodents (excluding dormice) overlapped highly with mesocarnivores in all scenarios, but the overlap was intermediate with the Iberian lynx (Fig. 4). We only found significant differences for the pair marten-rodents outside breeding and outside lynx territories (Fig. 4). However, significant differences were found among the circadian activity of carnivores (including lynx) and dormice outside breeding season in both predation risk scenarios, except for the pair marten-dormouse inside lynx territories (Fig. 4). Contrastingly, during the breeding season, we found no significant differences among the temporal patterns of mesocarnivores and dormice for any predation risk scenario and the same result was found for the pair lynx-dormouse during this period (Fig. 4).

### Spatio-temporal segregation among carnivores

**Figure 5:**
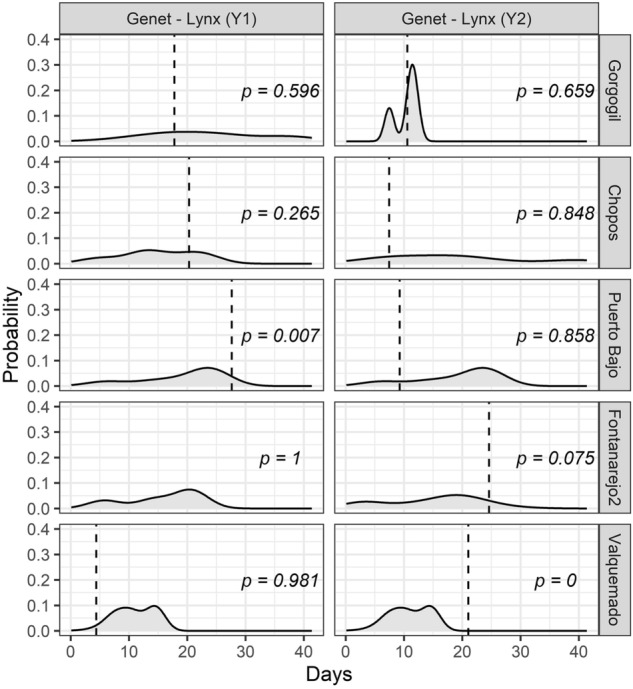
Spatio-temporal segregation patterns of common genets respect to the Iberian lynx in each locality and year. The vertical dashed lines represent the observed median minimum time-to-encounter between a lynx and a genet and the shaded grey area shows randomly simulated times-to-encounter. The p-values represents the proportion of randomly generated times-to-encounter values that are greater than the observed time-to-encounter.

Inside Iberian lynx territories, the proportion of visits of the common genet at camera sites without Iberian lynx ranged from 0 to 93% across localities in the first year (Mean ± SE: 47.58% ± 0.18) and from 40 to 93% in the second year (68.96% ± 0.09) (Supplementary Appendix S3; Table S6). Lynx and genets were detected simultaneously in fewer than 8% of the camera-traps at each time interval whereas genet and lynx co-occurred exclusively at up to 36% and 14% of the cameras, respectively (Supplementary Appendix S3: Table S7). The genet showed a notable avoidance behaviour mainly in the first year, reflected by an average time-to-encounter with lynx of 18.36 days (SE = 1.80) but also in the second year with 14.43 days (SE = 1.59) (Fig. 5). For the stone marten, we only detected one record spatially overlapping with lynx, while the 99.95% of the visits occurred in cameras where the lynx was not recorded, so that was impossible to calculate time-to-encounter.

### Time-to-encounter and spatial encounter probability

**Figure 6:**
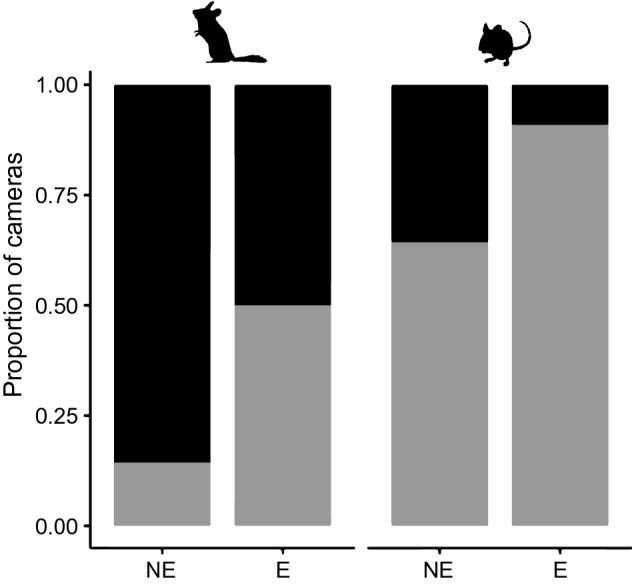
Proportion of camera-traps with presence of common genet inside lynx territories which in occurred an encounter with an Iberian lynx (E) or not (NE). Black colour shows the proportion of cameras with absence of micromammals and grey colour shows presence of micromammals. The first silhouette refers to the garden dormouse (*E. quercinus*) and the second silhouette to other rodents (*A. sylvaticus, M. spretus* and *R. rattus*).

We found that the median time interval between a visit of lynx and the following visit of a genet was not significantly affected by genet visitation rate and micromammal availability at camera level (Table 1). However, we found that the spatial encounter probability between lynx and genets was positive and significantly related to the presence of prey (both of garden dormouse and other rodents) (Table 1, Fig. 6).

## Discussion

**Figure 7:**
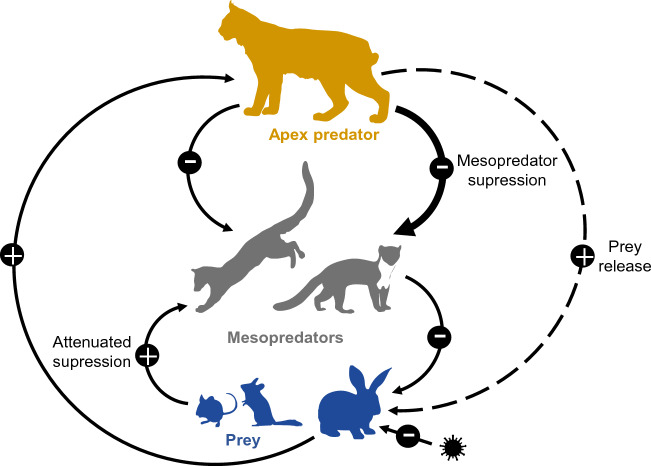
Direct (solid lines) and indirect trophic interactions (dashed lines) in the Mediterranean forest ecosystem. Trophic levels of the network are linked by top-down (descendent arrows) and bottom-up (ascendant arrows) forces which regulates the trophic web. Plus and minus symbols represent a positive or negative effect, respectively, and width of line indicates the relative strength of the interaction. Epizootic diseases (virus silhouette) determine the distribution of the European rabbit (*O. cuniculus*), the main prey of the Iberian lynx (*L. pardinus*) which is the apex predator in this ecosystem. Lynx controls mesopredator abundances and releases prey (rodents and rabbits) from their predation pressure. However, the suppression of the common genet (*G. genetta*) is lower compared to suppression of the stone marten (*M.s foina*) possibly attenuated due to its more specialized diet in micromammals.

Our natural experiment in Southern Spain reveals that competitive intra-guild interactions among carnivores can be relaxed by bottom-up forces in a scenario of apex predator restoration in Mediterranean ecosystems (see Fig. 7). The largest predator in the area, the Iberian lynx, exerted a strong top-down control on absolute abundances of two sympatric mesopredators with a high dietary overlap but different trophic plasticity. However, the suppression strength and the spatio-temporal avoidance strategies developed by subordinate predators markedly varied among species, which could lead to contrasting ecological implications for the ecosystem functioning. Although previous studies have documented the negative impact of the Iberian lynx on relative mesopredator abundances^27,33^, our study is pioneer on estimating the impact of an apex predator on the absolute mesopredator densities^26,34^. We documented for the first time the impact of Iberian lynx on common genet abundance and provided one of the most accurate density estimates for stone marten in Mediterranean ecosystems, which seems to be the species most sensitive to the Iberian lynx. Moreover, by using spatially explicit capture-recapture methods to estimate absolute abundance of mesocarnivores, we likely avoid bias which could lead us to underestimate movements and hence overestimate density.

The recovery of the Iberian lynx in our study area is recent and still spatially heterogeneous, primarily due to the patchy distribution of its main prey, the wild rabbit, which is in turn connected with localized epizootic eruptions leading to frequent local extinctions of rabbits^35^. Where the lynx was absent, the density of two sympatric mesopredators, the common genet and the stone marten, was up to 10-times larger than inside lynx territories. Similar mesopredator suppression has been shown by several apex predators across the world, such as the grey wolf, the dingo or the Eurasian lynx^13,36^. However, the strength of this top-down regulation differed considerably between the two mesopredator species, despite their similar size, diet and habitat use^30,37^. A narrower trophic niche of genets compared to martens could play a key role in dampening suppression and thus favouring coexistence with the apex predator^17,19^. Another potential contributing factor to explain this species-specific suppression could be different behavioural mechanisms to avoid agonistic encounters or tolerance to predation risk. For example, both genets and martens are able to climb trees easily to escape from a lynx but the perception of predation risk may be variable among habitats or species^37^. Stone martens use the three-dimensional space (i.e. movements above ground in tree crowns) less frequently than other sympatric species such as the pine marten (*Martes martes*)^38^, which could increase the likelihood of agonistic encounters with a lynx. This habitat-use segregation could also occur in respect to the genet, but fine-scale ecological studies on habitat use of these sympatric species are still insufficient for a comprehensive understanding of species-specific anti-predatory responses.

Although previous research has revealed that several species can modify their temporal activity patterns according to factors such as predation risk or human disturbance^22,39^, the circadian rhythms of genets and martens were predominately nocturnal and consistent among predation risk scenarios and seasons^23,40^. However, these mesocarnivores showed temporal segregation with the Iberian lynx, in contrast to Sarmento et al.^33^ findings for a more diurnal and adaptable mesopredator, the red fox. Thus, temporal segregation among subordinate carnivores and larger predators is often a result of an evolutionary adaptation to resource and habitat partitioning^20,23,41^.

As we expected for a micromammal specialist consumer, genets synchronized their activity patterns with rodents in all scenarios but did not so with rabbits^42^. Small mammals offer energetically richer food intake than other resources, such as fruits^43^, but due to the scarce abundance of rabbits in our study area, this prey seems to be less preferred by generalists^44^. In contrast, stone marten activity overlapped highly with small mammals only inside lynx territories and during the breeding season, when the availability of prey was maximum. Breeding coincides with the spring, when the garden dormouse comes out from its winter lethargy and increases activity^45^. Bakaloudis et al.^46^ also found an animal-type prey specialization by stone martens in spring, contrasting with the general tendency to consume fruits during winter in Mediterranean ecosystems. Thus, several pieces of evidence indicate that martens are shifting their activity rhythms and likely their diet according to prey availability, as was already observed in several other facultative predators^47^. However, our results on behaviour of martens under predation risk must be taken with caution because it may be a consequence of the limited amount of records of stone marten inside lynx territories (11 detections).

The higher dormice availability during the breeding season could be increasing the tolerance of genets to predation risk^29,42^, since temporal partitioning with lynx did not occur. Despite the general synchronized activity patterns found among genets and other rodent species, they only overlapped highly with dormice during the breeding season, and the same pattern was found for the Iberian lynx. Although the Iberian lynx is a rabbit-specialist, their low temporal overlap with this prey species could be explained because lynx is complementing its diet with the abundant garden dormouse^48^, especially when reproductive females are breeding and would benefit from additional, easy to catch prey, as was also observed in the Eurasian lynx^49^.

Genets and martens are behaviourally and ecologically similar and could be expected to develop analogous strategies to cope with larger predators. However, the stone marten almost disappeared from the Iberian lynx territories, which is in accordance with the strong avoidance patterns found by Monterroso et al.^17^ and Virgós et al.^50^. In contrast, the common genet co-existed spatially with the apex predator in low density by developing fine-scale spatio-temporal mechanisms^18,33^, similarly to observations of Jiménez et al.^26^ for other mesocarnivores following Iberian lynx reintroduction. Although previous studies described a high spatial segregation of genets in respect to lynx home ranges^17,26,27^, we found a surprising number of detections of genets in cameras where the lynx appeared (circa 50%), probably due to the higher abundance of genets and/or the greater forestry cover and refuge availability in our study area compared to other Mediterranean forests.

Bottom-up effects can also regulate the spatiotemporal coexistence patterns among carnivores^17–19,29^. Similarly to previous research found for other prey^26,51^, a reduced overall predation pressure inside lynx territories seemed to benefit small mammal abundances in our study area. A more specialized diet on rodents probably led some genets to persist inside lynx ranges in spite of the risk of being killed. Martens however were able to feed on a wider spectrum of food resources available in non-risky nearby areas. Encounters among genets and lynx were more likely when rodent availability was higher locally, what may suggest that both predators are taking advantage of the abundance of prey or genets visiting these food hotspots are less cautious because of high motivation to forage there, as predicted by the optimal foraging theory^31^.

Considering the strong lynx suppression of stone marten abundance, the impact on its spatial distribution patterns likely has indirect community level impacts. Carnivores are effective seed dispersers of many fleshy-fruited plants, dispersing large numbers of seeds to long distances but showing functional diversity among species^52,53^. Fruit seeds are found in up to 94% scats of stone marten, contrasting with the more specialised genet diet on micromammals^32,54^. Therefore, a suppressed abundance of key frugivorous carnivores as martens could trigger cascading effects for plants by limiting seed dispersal inside lynx territories^55–57^. However, the exclusion of stone martens from rodent-rich patches inhabited by lynx, could impact body condition and fitness^43^ and shift diet composition of marten population towards higher consumption of fruits^58^, triggering a two-told effect on the ecosystem functioning by limiting seed dispersal inside lynx territories but boosting it outside. Thus, the patchy lynx distribution and the paired suppression of mesopredators as a consequence of spatially variable abundance of lynx main prey, the rabbit, could be contributing to build a mosaic ecosystem structure where apex predators, mesopredators and prey have variable local abundances and connected ecological roles (Fig. 7).

Our findings suggest that Iberian lynx reintroduction or recovery could reverse a potential mesopredator release scenario in areas where this apex predator was lost or severely reduced, but the strength of this top-down effect in the trophic web is species-specific. The most diet-flexible mesopredator, the stone marten, is drastically reduced by the apex predator and shows high spatial and temporal segregation with the apex predator. Nevertheless, the mesocarnivore with a narrow trophic niche breadth, the common genet, co-exists spatially in reduced density with the Iberian lynx and also overlaps temporally during the breeding season. Furthermore, bottom-up forces also seem to be important in the trophic web by dampening the mesopredator suppression and facilitating coexistence. The contrasting suppression found for two sympatric mesocarnivores may alter important ecosystem functions and processes involving them. Thus, conservation practitioners should consider to promote a locally-patchy distribution of the Iberian lynx through the landscape because it may result in balanced net effects for different trophic levels at a regional scale. Further future detailed studies are essential to assess how apex predator reintroductions are restoring the ecosystem functioning prior to their extinction. This knowledge will lead to more effective conservation programs of carnivores by taking evidence-based decisions and a better understanding on the working of the ecosystem at community level.

## Methods

### Study area and target species

Our study was conducted in Sierra de Andújar Natural Park (Fig. 1). The annual average temperature (~ 18 °C) and rainfall (~ 700 mm) are typical for Mediterranean climate areas (data from Andújar meteorological station, AEMET Opendata, 2020). The altitude ranges between 400 and 800 m. Vegetation is dominated by Mediterranean shrubland and an arboreal stratum of holm oak (*Quercus ilex*). Extensive private and public lands compound the Natural Park, where hunting of large wild game ungulates is frequent, but small game hunting and predator control is rare or absent.

The Iberian lynx is the largest carnivore inhabiting the study area (6.1–15.9 kg^59^). Sierra de Andújar NP was one of the last refuge for this species in the 1990’s and 2000s when less than 100 individuals were left in the wild^59^. Despite the recovery of Iberian lynx populations in the last 20 years, lynx home ranges are heterogeneously distributed throughout the Natural Park (Fig. 1) and this feline is still absent in many lands as consequence of the lack of its main prey, the European rabbit (*Oryctolagus cuniculus*)^59^.

Nine wild mammal mesocarnivores inhabit the study area, including the red fox (*Vulpes vulpes)*, the stone marten, the common genet and the Eurasian badger (*Meles meles)*. Given that we aim to assess the suppression effect of top-order predator on mesopredator abundances by using SCR methods which require individual recognition and a high recapture rate^60^, we focussed this study on the common genet and the stone marten because they are abundant mesocarnivores with easily identifiable coat patterns (see Supplementary Appendix S1). They are sympatric carnivores of similar size (around 1 kg), habitat and food requirements but markedly differ in their trophic plasticity^32,37^. Small mammals are their main prey, followed by birds, fruits and invertebrates in decreasing order of importance^32^. Rabbits also represent a selected prey by these mesocarnivores when reach high abundances^30^. However, the stone marten is more adaptable to food availability, feeding on fruits more often in comparison with the more specialized diet of the genet, which is mainly focused on micromammals^32,54^. Thus, to assess the importance of prey availability on coexistence among predators, we focused on the most abundant small mammal species that inhabit our study area, the European rabbit, the garden dormouse (*Eliomys quercinus*), the wood mouse (*Apodemus sylvaticus*), the Algerian mouse (*Mus spretus*) and the black rat (*Rattus rattus*) (see Supplementary Appendix S1: Fig. S3).

### Experimental design and camera-trap data collection

We carried out a camera-trapping survey between October and May of 2018–19 and 2019–20 (Supplementary Appendix S3: Table S4). We used an experimental design comparing five localities with territorial presence of Iberian lynx and five localities out of lynx distribution range as controls with the same habitat characteristics (Fig. 1). We selected these localities based on our previous long-term research in the study system and following the recommendations of the wildlife monitoring program of the Natural Park. Control localities confirmed absence of reproduction and territorial lynx pairs, mostly due to the absence or scarcity of its main prey, the wild rabbit. Localities with lynx presence were distributed along the lynx distribution area shown by the IUCN Red List of Threatened Species^61^ (Fig. 1) and had verified lynx reproduction in the previous five years. These localities were considered spatially independent because they were situated far enough apart (Mean ± SD = 7.9 ± 3.0 km; Fig. 1) in order to absence localities kept out of the nearest Iberian lynx home range and presence localities included a territory of a different lynx couple (average lynx home range radius in our study area is 1.62 km)^62^.

We placed 120 camera traps (12 in each locality; Fig. 1) the first year and we set them again at the same locations the second year (Scoutguard SG562-C; white led). Cameras were placed at the height of 40–60 cm above the ground, operated 24 h/day and were programmed with a trigger delay of 1 s, to take three consecutive images. Each camera worked an average of 44 days and reached an overall effort of 10,644 trap-days (see Supplementary Appendix S3: Table S4 for more details). At each camera station, we placed under a small rock a hygienic tampon soaked with Iberian lynx urine (collected from several captive individuals in breeding centres, males and females mixture) and a sardine can on the top of a wood stick at a height of 60 cm to 1.5 m from the camera (see photographs in Supplementary Appendix S1). The use of lures has been proved not to affect abundance estimates or temporal activity in camera-trapping studies and is a widely-used strategy to improve detectability in SCR models^63,64^. Lynx urine is one of the most effective attractants of mesocarnivores^65^ and sardines are useful to keep the animals long enough in front of the camera-trap to take several images, thus improving the likelihood of obtaining individual identification. Two experts successfully individually identified predators based on unique coat patterns by consensus in 70% of lynx detections, 93% for stone marten and 87% for genets inside lynx territories and 82% for martens and 92% for genets outside lynx range (see Supplementary Appendix S1 for more details). Detection of small-mammal prey species was also improved due to the use of lure, because most of them climbed up to the top of the stick where sardines were placed, making it easier to identify the species (see Supplementary Appendix S1 Fig. S3). We classified small mammals into three categories: rabbit, garden dormouse and other rodents which grouped rodent species of similar morphology, such as the wood mouse, the Algerian mouse and the black rat. We considered an event as independent when we identified the individual as different from the previous recording or the time interval between successive images was at least 30 min for non-identified or the same individuals^66^. Within each locality, camera-traps were spaced an average distance of 683 ± 145.61 m (Mean ± SD) from the nearest camera. We set this distance because it was lower than 2σ (σ is the movement parameter^67^) of the carnivore species with the smallest averaged home range size (381 ha for the stone marten^68^) following Royle et al.^69^ recommendations. We covered a surveyed surface of ca. 900 ha in each locality, enough for the mesopredator with the biggest home range size, in our case the genet (781 ha^70^).

### Abundance estimates

We estimated the density (individuals/km^2^) of genet and stone marten in each locality with and without Iberian lynx presence for each study year by using a capture-recapture database of identified records. Population was considered demographically closed because density estimates were carried out with data from October to May, out of the main juvenile dispersal period of these species which occurs during late summer and early autumn^71^. We used spatial capture-recapture (SCR) models^72^ to estimate capture probability as a decreasing function of the distance between activity centres and detectors (camera-traps) and calculated density estimates^60^. In our study we assume the capture history ${y}_{ij}$ are mutually independent outcomes of a binomial random variable, which we express as:

1 $${y}_{ij}\sim Binomial\left(K,{\lambda }_{ij}\right).$$

The link function between the location of detectors and the activity centres of the individuals follows a half-normal distribution:

2 $${\lambda }_{ijk}={\lambda }_{0}exp\left(\frac{-{d}_{ij}^{2}}{{2\sigma }^{2}}\right),$$

where ${d}_{ij}$ is the Euclidean distance between the activity centre for each individual and camera-trap location, $\sigma$ is the scale parameter from half normal distribution (that described the movement), and ${\lambda }_{0}$ is the baseline encounter rate. We implemented spatially-explicit models in a Bayesian framework using R (R Core Team, 2018) and Nimble package^73,74^ similarly to Jiménez et al.^26^. In order to get better parameter estimates for the stone marten, we used models with shared movement parameter sigma ($\sigma$) and baseline detection rate (${\lambda }_{0}$) among localities with lynx presence and lynx absence because we recorded few spatial re-captures in localities with lynx^26,69,75^. For common genets, we estimated $\sigma$ and ${\lambda }_{0}$ separately for localities with and without lynx. Posterior probabilities were calculated using 3 independent MCMC chains. For stone marten we used 55,000 iterations each, and a burn-in of 5000 iterations. For genet we used 160,000 iterations with an initial 10,000 iterations as a burn-in, and thinned the remainder by 5. We confirmed model convergence by examining trace plots (see Supplmentary Appendix S2; Figs. S2–S4) and ensuring that the potential scale reduction factor (R-hat) statistic for each parameter was < 1.1^76^.

### Temporal overlap

Temporal analysis was carried out by generating the kernel density estimation of the activity patterns of lynx, genet, stone marten and prey species (rabbits, garden dormice and other rodents) using the overlap package^66^. We carried out these analyses separately for the breeding season (i.e. denning period from March to June) and for the non-breeding season (excluding the detections from March to June; see Supplmentary Appendix S3: Table S5) of genets (Camps^71^), stone martens (Mangas 2007) and lynx^59^. We took this decision due to previous research showing effects of breeding behaviour on circadian rhythms of these predators^23^.

We calculated the overlap coefficients (Δ) of temporal activity among (1) lynx and genet and lynx and stone marten, (2) genet and marten inside and outside lynx range and (3) carnivores with prey species, considering only species with ≥ 10 detections (Supplmentary Appendix S3: Table S5). In line with Ridout and Linkie^66^, we used the estimator Δ_4_, which is recommended for large sample sizes ($\overline{n }$ ± SE = 1238.41 ± 214.22 detections; see Supplmentary Appendix S3: Table S5). The overlap coefficient varies from 0 (no overlap) and 1 (total overlap)^77^. We defined three overlap categories according the overlap values obtained from pairwise comparisons carried out with our data: Δ_4_ < 50th percentile was considered as “low overlap”; 50th < Δ_4_ < 75th as “moderate overlap”; and Δ_4_ > 75th as “high overlap”; being the 50th and 75th percentiles for our overlapping coefficients 0.8 and 0.9, respectively^23^. Confidence intervals for the overlap coefficients were obtained as percentile intervals from 1000 bootstrap samples. Then, we compared the activity patterns of each species pairs by using Watson’s two-sample test of homogeneity (U^2^) for circular data^78^.

### Spatio-temporal segregation

We applied two methods to assess spatio-temporal segregation only among lynx and genets, because the stone marten was recorded almost exclusively in cameras where lynx did not occur (see Table S7). Firstly, we built a matrix of detections per hour of the daily cycle for each camera-trap, locality and year for both species. Then, we calculated the averaged proportion of cameras with co-occurrence and exclusive occurrence for each species along the diel cycle. Confidence intervals were calculated by empirical bootstrapping^79^. Secondly, we estimated time-to-encounter between a lynx detection and the following genet detections in each locality and year using the multi-response permutation procedures showed by Mielke et al.^80^. We calculated the probability of time-to-encounter by comparing the median observed value with the simulated expected distribution (1000 simulations) and obtaining the proportion of randomly generated values that are greater than the observed time-to-encounter. A higher value of observed time-to-encounter in comparison to the expected value meant avoidance and a lower value indicated aggregation. We carried out these analyses by using the R codes provided by Karanth et al.^18^.

### Modelling

We fitted Generalized Linear Mixed Models (GLMM) to evaluate (1) the top-down effect of Iberian lynx presence and the bottom-up effect of the prey relative abundance on the abundance of mesocarnivores, (2) whether the time-to-encounter between genets and lynx was related with prey and genet visitation rate and (3) whether the encounter probability between genet and lynx was affected by the availability of prey (see Table 1). The number of captures by 100 cam-days were used to calculate visitation rates at camera level and relative abundance indices (RAI) at locality level.

The presence/absence of Iberian lynx was included as a fixed factor for the response density of mesocarnivores and we added up second-order interactions among this variable and the fixed factors prey species (rabbits, garden dormice and other rodents) and carnivore species (stone marten and common genet) and the covariates prey relative abundance (RAI) and carnivore density. The locality was used as a random factor. We used gaussian errors and applied log transformation. Regarding time-to-encounter, the response variable was the median time to encounter in days from every visit of a lynx up to a genet visit in each camera and the fixed effects were the genet and prey visitation rate and its interaction with the micromammal species (garden dormice and other rodents) at camera level. Camera station was included as a random effect and Poisson errors were used. We excluded the rabbit of these analysis because it does not seem to be an important prey for genets based on our temporal overlap results. We upper-limited the garden dormouse visitation rate by camera to 200 because we detected five outliers which biased our results. Lastly, for the spatial encounter probability among genet and lynx, we used a binomial response of encounter/non-encounter in each camera and we included the presence/absence of the garden dormouse and other rodents as fixed effects and the camera site as random effect.

We calculated the marginal (contribution of fixed effects) and conditional (both fixed and random effects) coefficient of determination (pseudo-R^2^) for all proposed mixed models^81^. We used R base functions (version 3.6.1; R Core Team 2019) and specialized packages (lme4 v.1.1–21 for GLMMs^82^; MuMIn v.1.9.5 for pseudo-R^2^^83^).

### Ethics statement

Camera-trapping surveys were approved by the Sierra de Andújar Natural Park, Spain, under permit PNSierraAndújar/300/2018 in accordance with animal welfare regulations. All methods are reported following the ARRIVE guidelines.

## Supplementary Information

Supplementary Information.

## Data Availability

The datasets generated and/or analyzed during the current study are not publicly available due the study species are protected and this information could be sensitive, but are available from the corresponding author on reasonable request.

## Code availability

Nimble-R code is available in Supplementary Information (S4–S7).

## References

[1] Alston JM (2019). Reciprocity in restoration ecology: When might large carnivore reintroduction restore ecosystems?. Biol. Conserv..

[2] Ripple WJ, Beschta RL (2012). Large predators limit herbivore densities in northern forest ecosystems. Eur. J. Wildl. Res..

[3] Estes JA, Duggins DO (1995). Sea otters and kelp forests in Alaska: Generality and variation in a community ecological paradigm. Ecol. Monogr..

[4] Schmitz OJ, Beckerman AP, O’Brien KM (1997). Behaviorally mediated trophic cascades: Effects of predation risk on food web interactions. Ecology.

[5] Power ME (1992). Top-down and bottom-up forces in food webs: Do plants have primacy. Ecology.

[6] Travers T, Lea MA, Alderman R, Terauds A, Shaw J (2021). Bottom-up effect of eradications: The unintended consequences for top-order predators when eradicating invasive prey. J. Appl. Ecol..

[7] Stoessel M, Elmhagen B, Vinka M, Hellström P, Angerbjörn A (2019). The fluctuating world of a tundra predator guild: bottom-up constraints overrule top-down species interactions in winter. Ecography (Cop.).

[8] Wolf C, Ripple WJ (2018). Rewilding the world ’s large carnivores. R. Soc. Open Sci..

[9] Krofel M, Jerina K (2016). Mind the cat: Conservation management of a protected dominant scavenger indirectly affects an endangered apex predator. Biol. Conserv..

[10] Prugh LR, Sivy KJ (2020). Enemies with benefits: Integrating positive and negative interactions among terrestrial carnivores. Ecol. Lett..

[11] Caro TM, Stoner CJ (2003). The potential for interspecific competition among African carnivores. Biol. Conserv..

[12] Linnell JDC, Strand O (2000). Interference interactions, co-existence and conservation of mammalian carnivores. Divers. Distrib..

[13] Newsome TM (2017). Top predators constrain mesopredator distributions. Nat. Commun..

[14] Crooks K, Soulé M (1999). Mesopredator release and avifaunal extinctions in a fragmented system. Nature.

[15] Schoener TW (1974). Resource partitioning in ecological communities. Science.

[16] Fedriani JM, Fuller TK, Sauvajot RM, York EC (2000). Competition and intraguild predation among three sympatric carnivores. Oecologia.

[17] Monterroso P, Díaz-Ruiz F, Lukacs PM, Alves PC, Ferreras P (2020). Ecological traits and the spatial structure of competitive coexistence among carnivores. Ecology.

[18] Karanth KU (2017). Spatio-temporal interactions facilitate large carnivore sympatry across a resource gradient. Proc. R. Soc. B Biol. Sci..

[19] Ferreiro-Arias I, Isla J, Jordano P, Benítez-López A (2021). Fine-scale coexistence between Mediterranean mesocarnivores is mediated by spatial, temporal, and trophic resource partitioning. Ecol. Evol..

[20] Di Bitetti MS, De Angelo CD, Di Blanco YE, Paviolo A (2010). Niche partitioning and species coexistence in a Neotropical felid assemblage. Acta Oecol..

[21] Carvalho JC, Gomes P (2004). Feeding resource partitioning among four sympatric carnivores in the Peneda-Gerês National Park (Portugal). J. Zool..

[22] Gil-Sánchez JM, Mañá-Varela B, Herrera-Sánchez FJ, Urios V (2021). Spatio-temporal ecology of a carnivore community in middle atlas NW of Morocco. Zoology.

[23] Monterroso P, Alves PC, Ferreras P (2014). Plasticity in circadian activity patterns of mesocarnivores in Southwestern Europe: Implications for species coexistence. Behav. Ecol. Sociobiol..

[24] Gallagher AJ, Creel S, Wilson RP, Cooke SJ (2017). Energy landscapes and the landscape of fear. Trends Ecol. Evol..

[25] Sergio F, Hiraldo F (2008). Intraguild predation in raptor assemblages: A review. Ibis.

[26] Jiménez J (2019). Restoring apex predators can reduce mesopredator abundances. Biol. Conserv..

[27] Palomares F, Ferreras P, Fedriani JM, Delibes M (1996). Spatial relationships between Iberian lynx and other carnivores in an area of south-western Spain. J. Appl. Ecol..

[28] Wooster EIF, Ramp D, Lundgren EJ, O’Neill AJ, Wallach AD (2021). Red foxes avoid apex predation without increasing fear. Behav. Ecol..

[29] Santos F (2019). Prey availability and temporal partitioning modulate felid coexistence in Neotropical forests. PLoS ONE.

[30] Barrientos R, Virgós E (2006). Reduction of potential food interference in two sympatric carnivores by sequential use of shared resources. Acta Oecol..

[31] MacArthur RH, Pianka ER (1966). On optimal use of a patchy environment. Am. Nat..

[32] López-Martín, J. M. Comparison of feeding behaviour between stone marten and common genet: living in coexistence. *Martes Carniv. Communities* 137–155 (2006).

[33] Sarmento P (2021). Adapt or perish: How the Iberian lynx reintroduction affects fox abundance and behaviour. Hystrix Ital. J. Mammal..

[34] Forsyth DM, Ramsey DSL, Woodford LP (2019). Estimating abundances, densities, and interspecific associations in a carnivore community. J. Wildl. Manag..

[35] Monterroso P (2016). Disease-mediated bottom-up regulation: An emergent virus affects a keystone prey, and alters the dynamics of trophic webs. Sci. Rep..

[36] Ritchie EG (2012). Ecosystem restoration with teeth: What role for predators?. Trends Ecol. Evol..

[37] Santos-Reis M (2004). Relationships between stone martens, genets and cork oak woodlands in Portugal. Martens Fish. Hum.-Altered Environ. Int. Perspect..

[38] Goszczyński J, Posłuszny M, Pilot M, Gralak B (2007). Patterns of winter locomotion and foraging in two sympatric marten species: *Martes martes* and *Martes foina*. Can. J. Zool..

[39] Díaz-Ruiz F, Caro J, Delibes-Mateos M, Arroyo B, Ferreras P (2016). Drivers of red fox (*Vulpes vulpes*) daily activity: Prey availability, human disturbance or habitat structure?. J. Zool..

[40] Zanón Martínez JI, Seoane J, Kelly MJ, Sarasola JH, Travaini A (2021). Assessing carnivore spatial co-occurrence and temporal overlap in the face of human interference in a semi-arid forest. Ecol. Appl..

[41] Allen ML, Sibarani MC, Utoyo L, Krofel M (2020). Terrestrial mammal community richness and temporal overlap between tigers and other carnivores in Bukit Barisan Selatan National Park Sumatra. Anim. Biodivers. Conserv..

[42] Vilella M, Ferrandiz-Rovira M, Sayol F (2020). Coexistence of predators in time: Effects of season and prey availability on species activity within a Mediterranean carnivore guild. Ecol. Evol..

[43] Santos N (2020). Protein metabolism and physical fitness are physiological determinants of body condition in Southern European carnivores. Sci. Rep..

[44] Ferreras P, Travaini A, Cristina Zapata S, Delibes M (2011). Short-term responses of mammalian carnivores to a sudden collapse of rabbits in Mediterranean Spain. Basic Appl. Ecol..

[45] Moreno S (1988). Reproduction of Garden Dormouse Eliomys quercinus lusitanicus, in southwest Spain. Mammalia.

[46] Bakaloudis DE, Vlachos CG, Papakosta MA, Bontzorlos VA, Chatzinikos EN (2012). Diet composition and feeding strategies of the stone marten (*Martes foina*) in a typical mediterranean ecosystem. Sci. World J..

[47] Pereira LM, Owen-Smith N, Moleón M (2014). Facultative predation and scavenging by mammalian carnivores: Seasonal, regional and intra-guild comparisons. Mamm. Rev..

[48] Gil-Sánchez JM, Ballesteros-Duperón E, Bueno-Segura JF (2006). Feed ing ecology of the Iberian lynx Lynx pardinus in east ern. Acta Theriol. (Warsz).

[49] Krofel M, Huber D, Kos I (2011). Diet of Eurasian lynx Lynx lynx in the northern Dinaric Mountains (Slovenia and Croatia). Acta Theriol. (Warsz).

[50] Virgós E, Baniandrés N, Burgos T, Recio MR (2020). Intraguild predation by the eagle owl determines the space use of a mesopredator carnivore. Diversity.

[51] Gordon CE, Feit A, Grüber J, Letnic M (2015). Mesopredator suppression by an apex predator alleviates the risk of predation perceived by small prey. Proc. R. Soc. B Biol. Sci..

[52] Draper JP, Young JK, Schupp EW, Beckman NG, Atwood TB (2022). Frugivory and seed dispersal by carnivorans. Front. Ecol. Evol..

[53] González-Varo JP, López-Bao JV, Guitián J (2013). Functional diversity among seed dispersal kernels generated by carnivorous mammals. J. Anim. Ecol..

[54] Virgós E, Llorente M, Cortés Y (1999). Geographical variation in genet (*Genetta genetta* L.) diet: A literature review. Mamm. Rev..

[55] Fedriani JM, Ayllón D, Wiegand T, Grimm V (2020). Intertwined effects of defaunation, increased tree mortality and density compensation on seed dispersal. Ecography (Cop.).

[56] Burgos T (2022). Predation risk can modify the foraging behaviour of frugivorous carnivores: Implications of rewilding apex predators for plant–animal mutualisms. J. Anim. Ecol..

[57] Escribano-Ávila G (2013). Spanish juniper gain expansion opportunities by counting on a functionally diverse dispersal assemblage community. Ecol. Evol..

[58] Gazzola A, Balestrieri A (2020). Nutritional ecology provides insights into competitive interactions between closely related Martes species. Mamm. Rev..

[59] Simón, M. A. et al. Diez años de conservación del lince ibérico, 326 (2012).

[60] Royle JA, Chandler RB, Sollmann R, Gardner B (2014). Spatial Capture-Recapture.

[61] Rodríguez, A. & Calzada, J. Lynx pardinus (errata version published in 2020). The IUCN Red List of Threatened Species 2015. 10.2305/IUCN.UK.2015-2.RLTS.T12520A174111773.en (Accessed 27 January 2023) (2015).

[62] Gil-Sánchez JM (2011). The use of camera trapping for estimating Iberian lynx (Lynx pardinus) home ranges. Eur. J. Wildl. Res..

[63] Gerber BD, Karpanty SM, Kelly MJ (2012). Evaluating the potential biases in carnivore capture-recapture studies associated with the use of lure and varying density estimation techniques using photographic-sampling data of the Malagasy civet. Popul. Ecol..

[64] Jiménez J, Díaz-Ruiz F, Monterroso P, Tobajas J, Ferreras P (2022). Occupancy data improves parameter precision in spatial capture–recapture models. Ecol. Evol..

[65] Ferreras P, DÍaz-Ruiz F, Monterroso P (2018). Improving mesocarnivore detectability with lures in camera-trapping studies. Wildl. Res..

[66] Ridout MS, Linkie M (2009). Estimating overlap of daily activity patterns from camera trap data. J. Agric. Biol. Environ. Stat..

[67] Jiménez J (2017). Estimating carnivore community structures. Sci. Rep..

[68] Genovesi P, Sinibaldi I, Boitani L (1997). Spacing patterns and territoriality of the stone marten. Can. J. Zool..

[69] Royle JA, Converse SJ (2014). Hierarchical spatial capture-recapture models: Modelling population density in stratified populations. Methods Ecol. Evol..

[70] Palomares F, Delibes M (1994). Spatio-temporal ecology and behavior of European genets in southwestern Spain. J. Mammal..

[71] Camps, D. Jineta - Genetta genetta. *En Encicl. Virtual los Vertebr. Españoles. Salvador. A., Barja, I. (Eds.). Mus. Nac. Ciencias Nat. Madrid. *https://www.vertebradosibericos.org/ (2017).

[72] Efford M (2004). Density estimation in live-trapping studies. Oikos.

[73] de Valpine P (2017). Programming with models: Writing statistical algorithms for general model structures with NIMBLE. J. Comput. Graph. Stat..

[74] NIMBLE Development Team. NIMBLE user manual (2017).

[75] Morin DJ, Waits LP, McNitt DC, Kelly MJ (2018). Efficient single-survey estimation of carnivore density using fecal DNA and spatial capture-recapture: A bobcat case study. Popul. Ecol..

[76] Gelman A (2013). Bayesian Data Analysis.

[77] Weitzman, M. S. *Measure of the Overlap of Income Distribution of White and Negro Families in the United States. Technical report N*^*o*^* 22* (1970).

[78] Jammalamadaka SR, Sengupta A (2001). Topics in Circular Statistics. Series on Multivariate Analyisis.

[79] Efron B, Tibshirani RJ (1994). An Introduction to the Bootstrap.

[80] Mielke PW, Berry KJ, Johnson ES (1976). Multi-response permutation proccedures for a priori classifications. Commun. Stat. Theory Methods.

[81] Nakagawa S, Johnson PCD, Schielzeth H (2017). The coefficient of determination R2 and intra-class correlation coefficient from generalized linear mixed-effects models revisited and expanded. J. R. Soc. Interface.

[82] Bates, D., Mächler, M., Bolker, B. M. & Walker, S. C. lme4: Linear mixed-effects models. *R Packag. version 1.1.21* (2020).

[83] Barton, K. Package “MuMIn: Multi-model inference” for R. *R Packag. Version 1.9.5* 45 (2013).

