# Supplementary material for: Top-down and bottom-up effects modulate species co-existence in a context of top predator restoration
Source: Sci Rep. 2023 Mar 13;13:4170. doi: 10.1038/s41598-023-31105-w (PMC10011582; doi:10.1038/s41598-023-31105-w)
Supplement: Supplementary file 1 — Supplementary Information. [file 41598_2023_31105_MOESM1_ESM.pdf]

Top-down and bottom-up effects modulate species co-existence in a context of top predator restoration

Appendix S1. Individual identification

Tamara Burgos[1]*, Javier Salesa[1], Jose María Fedriani[2,3], Gema Escribano-Ávila[4], José Jiménez[5], Miha Krofel[7], Inmaculada Cancio[1,6], Javier Hernández-Hernández[1,8], Javier Rodríguez-Siles[6] and Emilio Virgós[1]

[1]Área de Biodiversidad y Conservación, Departamento de Biología, Geología, Física y Química Inorgánica, Rey Juan Carlos University, Madrid, Spain

[2]Centro de Investigaciones sobre Desertificación CIDE, CSIC-UVEG-GV, Carretera de Moncada a Náquera, km 4,5. 46113 Moncada (Valencia), Spain

[3]Estación Biológica de Doñana (EBD – CSIC), Seville, Spain

[4]Biodiversity, Ecology and Evolution Department. Biological Science Faculty. Universidad Complutense de Madrid. Ciudad Universitaria, C/ José Antonio Novais 12, Madrid, Spain

[5]Instituto de Investigación en Recursos Cinegéticos (CSIC-UCLM-JCCM), 13071 Ciudad Real, Spain

[6]Asociación de Estudio y Conservación de Fauna Harmusch, C/San Antón 15, 1º 13580, Almodóvar del Campo, Ciudad Real, Spain

[7]Department for Forestry, Biotechnical Faculty, University of Ljubljana, Ljubljana, Slovenia.

[8]Road Ecology Lab, Department of Biodiversity, Ecology and Evolution, Faculty of Biology, Complutense University of Madrid, Madrid, Spain

**Correspondence author: tamara.burgos@urjc.es*

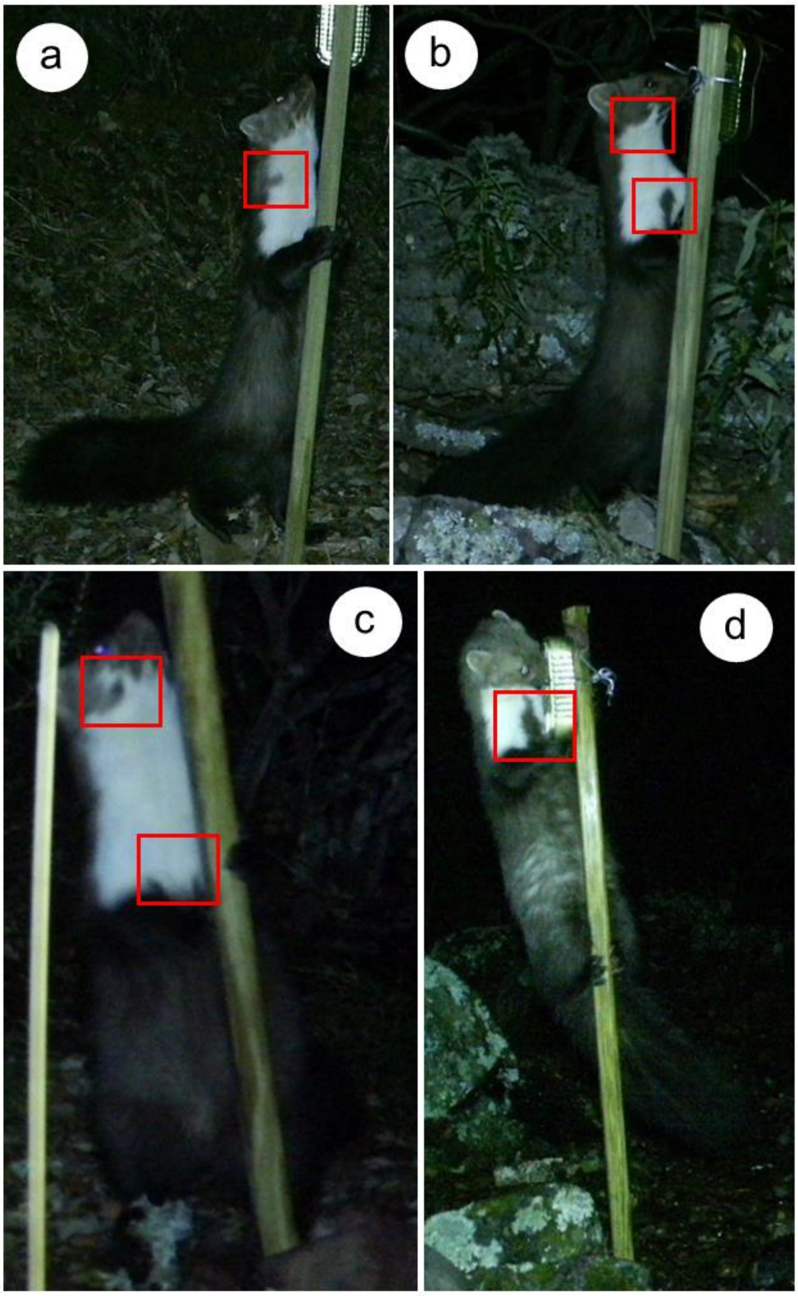

Figure S1. a-d show four different stone martens (*Martes foina*) individuals identified in the study based on distinctive fur marks (red boxes). The stone marten has a uniform coat pattern which makes this species particularly difficult to identify individually. Due to stone martens stood up to reach the bait at the top of the wooden sticks used in this study, we were able to identify successfully the most of the stone marten individuals because each individual presents several unique marks along the white throat.

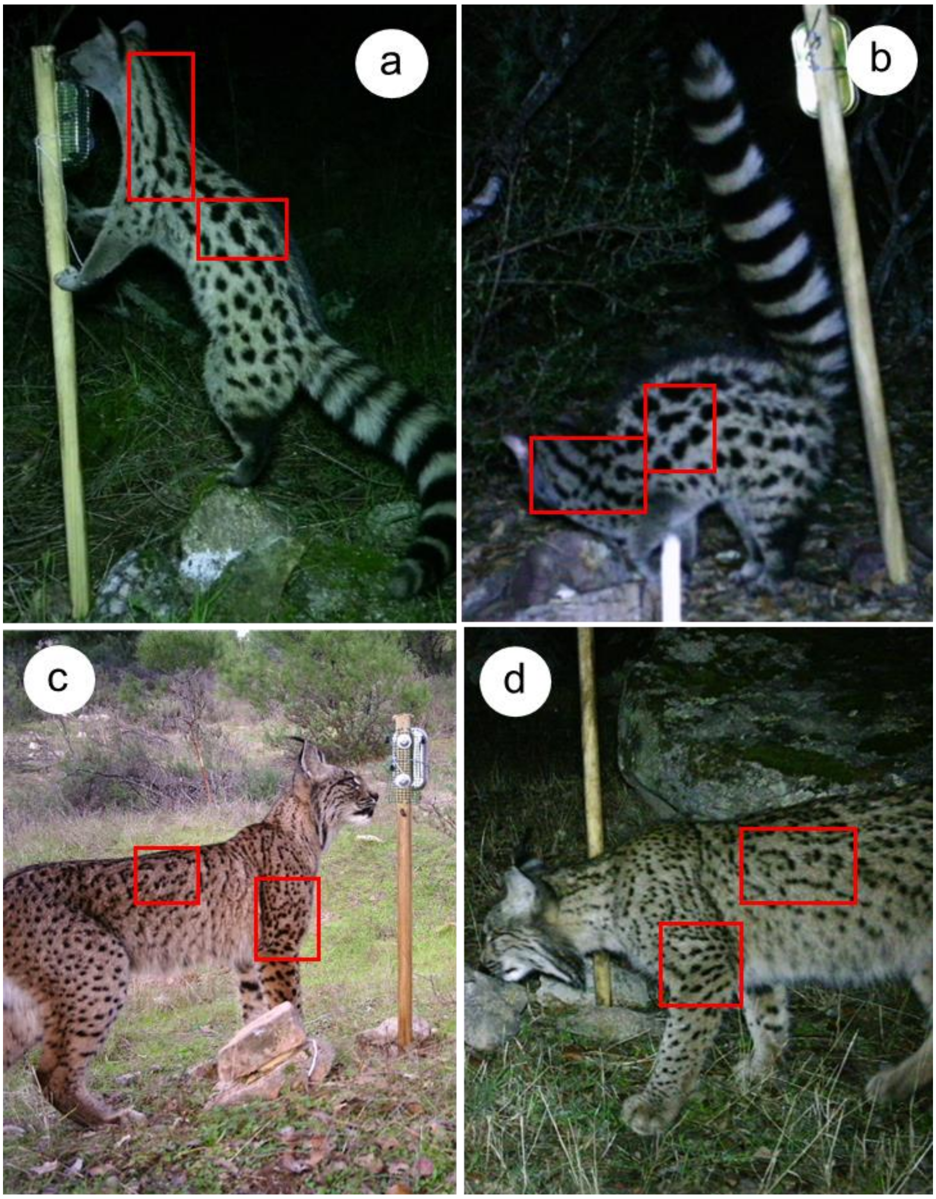

Figure S2. Spot patterns (red boxes) used for individual identification of the common genet *Genetta genetta* (ab) and the Iberian lynx *Lynx pardinus (cd)*. We needed to have photographs from the two sides of these species because the spot patterns vary. Thank we used alternative baits (sardine and lynx urine), we usually obtained more than one photograph of each individual in different positions during the same event, which allowed us to identify correctly the individuals.

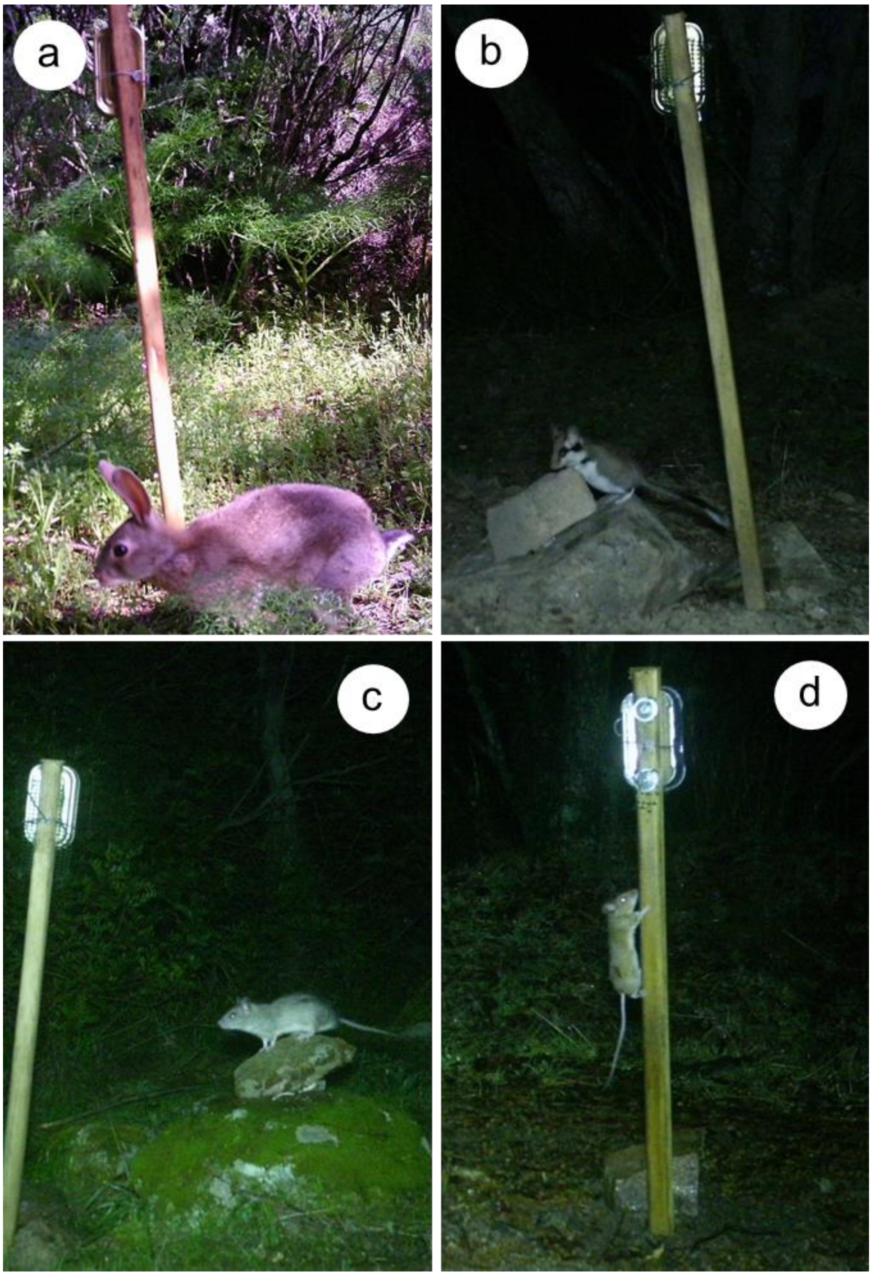

Figure S3. Photographs of the main small-mammal prey species of the studied carnivorous guild: a) European wild rabbit (*Oryctolagus cuniculus*), b) Garden dormouse (*Eliomys quercinus*) and other rodent species such as c) the black rat (*Rattus rattus*) or d) the wood mouse (*Apodemus sylvaticus*). Our camera-trapping scenario has been proved useful to get good-quality images of small mammals because in mostly occasions, they appeared on the top of the wood stick attracted by the lure.

Top-down and bottom-up effects modulate species co-existence in a context of top predator restoration

Appendix S2. Density estimates

Tamara Burgos[1]*, Javier Salesa[1], Jose María Fedriani[2,3], Gema Escribano-Ávila[4], José Jiménez[5], Miha Krofel[7], Inmaculada Cancio[1,6], Javier Hernández-Hernández[1,8], Javier Rodríguez-Siles[6] and Emilio Virgós[1]

[1]*Área de Biodiversidad y Conservación, Departamento de Biología, Geología, Física y Química Inorgánica, Rey Juan Carlos University, Madrid, Spain*

[2]*Centro de Investigaciones sobre Desertificación CIDE, CSIC-UVEG-GV, Carretera de Moncada a Náquera, km 4,5. 46113 Moncada (Valencia), Spain*

[3]*Estación Biológica de Doñana (EBD – CSIC), Seville, Spain*

[4]*Biodiversity, Ecology and Evolution Department. Biological Science Faculty. Universidad Complutense de Madrid. Ciudad Universitaria, C/ José Antonio Novais 12, Madrid, Spain*

[5]*Instituto de Investigación en Recursos Cinegéticos (CSIC-UCLM-JCCM), 13071 Ciudad Real, Spain*

[6]*Asociación de Estudio y Conservación de Fauna Harmusch, C/San Antón 15, 1º 13580, Almodóvar del Campo, Ciudad Real, Spain*

[7]*Department for Forestry, Biotechnical Faculty, University of Ljubljana, Ljubljana, Slovenia.*

[8]*Road Ecology Lab, Department of Biodiversity, Ecology and Evolution, Faculty of Biology, Complutense University of Madrid, Madrid, Spain*

Correspondence author: tamara.burgos@urjc.es

Table S1. Mean density (individuals/km^2) with 95% Bayesian credible intervals (BCI) estimated for mesocarnivores in each locality and year. The shared movement parameter (σ) estimated for stone marten was 0.56 (BCI: 0.51-0.61) in the first year and 0.65 (BCI: 0.60-0.70) in the second year. Genet sigma was 0.92 (BCI: 0.75-1.19) and 1.02 (BCI: 0.85-1.26) for the first year and 0.97 (BCI: 0.81-1.17) and 0.64 (BCI: 0.59-0.68) for the second year, in localities with lynx and without lynx respectively.

**Year**	**Lynx**	**Locality**	*G. genetta* **density**		*M. foina* **density**	
			Mean	BCI	Mean	BCI
1	Presence	Gorgogil	0.22	0.12-0.39	0.03	0.00-0.12
		Puerto Bajo	0.16	0.07-0.33	0.09	0.02-0.24
		Valquemado	0.08	0.03-0.19	0.03	0.00-0.13
		Fontanarejo2	0.16	0.07-0.32	0.03	0.00-0.14
		Chopos	0.17	0.08-0.32	0.03	0.00-0.13
	Absence	Lugar Nuevo	0.22	0.13-0.38	0.32	0.15-0.56
		Selladores	0.27	0.16-0.48	0.81	0.52-1.17
		Fontanarejo1	0.17	0.09-0.31	0.63	0.37-0.97
		Cereceda	0.19	0.10-0.35	0.38	0.18-0.68
		Risquillo	0.22	0.12-0.36	0.26	0.12-0.48
2	Presence	Gorgogil	0.18	0.09-0.31	0.02	0.00-0.09
		Puerto Bajo	0.04	0.01-0.12	0.07	0.01-0.18
		Valquemado	0.14	0.07-0.25	0.02	0.00-0.09
		Fontanarejo2	0.15	0.07-0.27	0.10	0.03-0.22
		Chopos	0.23	0.14-0.37	0.02	0.00-0.09
	Absence	Lugar Nuevo	0.64	0.42-0.92	0.20	0.10-0.36
		Selladores	0.51	0.33-0.76	0.66	0.45-0.95
		Fontanarejo1	0.54	0.33-0.81	0.47	0.28-0.72
		Cereceda	0.22	0.10-0.41	0.43	0.24-0.68
		Risquillo	0.40	0.23-0.63	0.17	0.07-0.32

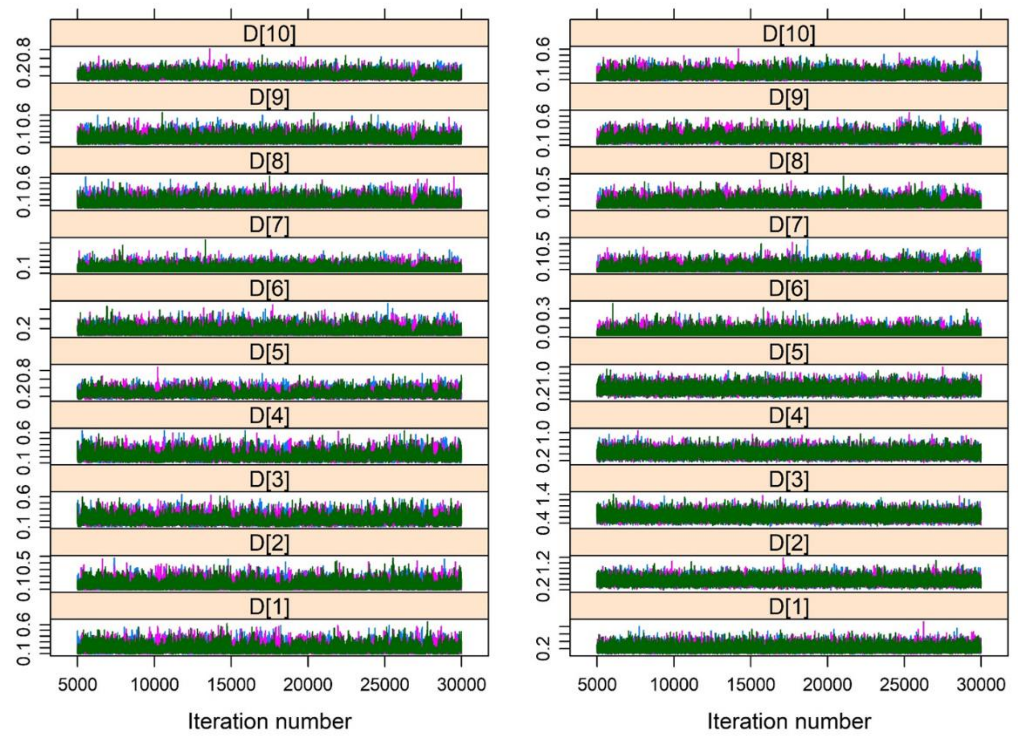

Figure S1. Convergence of Markov chains for the density of common genet *G. genetta* for the first (left) and the second study year (right) in the ten localities.

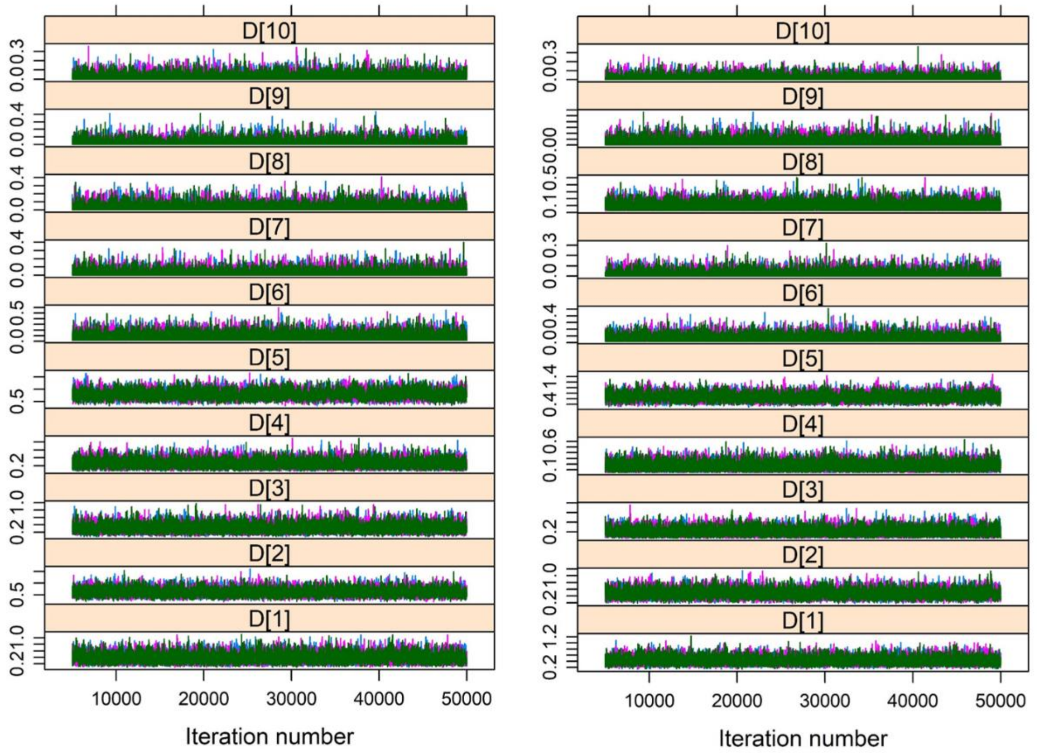

Figure S2. Convergence of Markov chains for the density of stone marten *M. foina* for the first (left) and the second study year (right) in the ten localities.

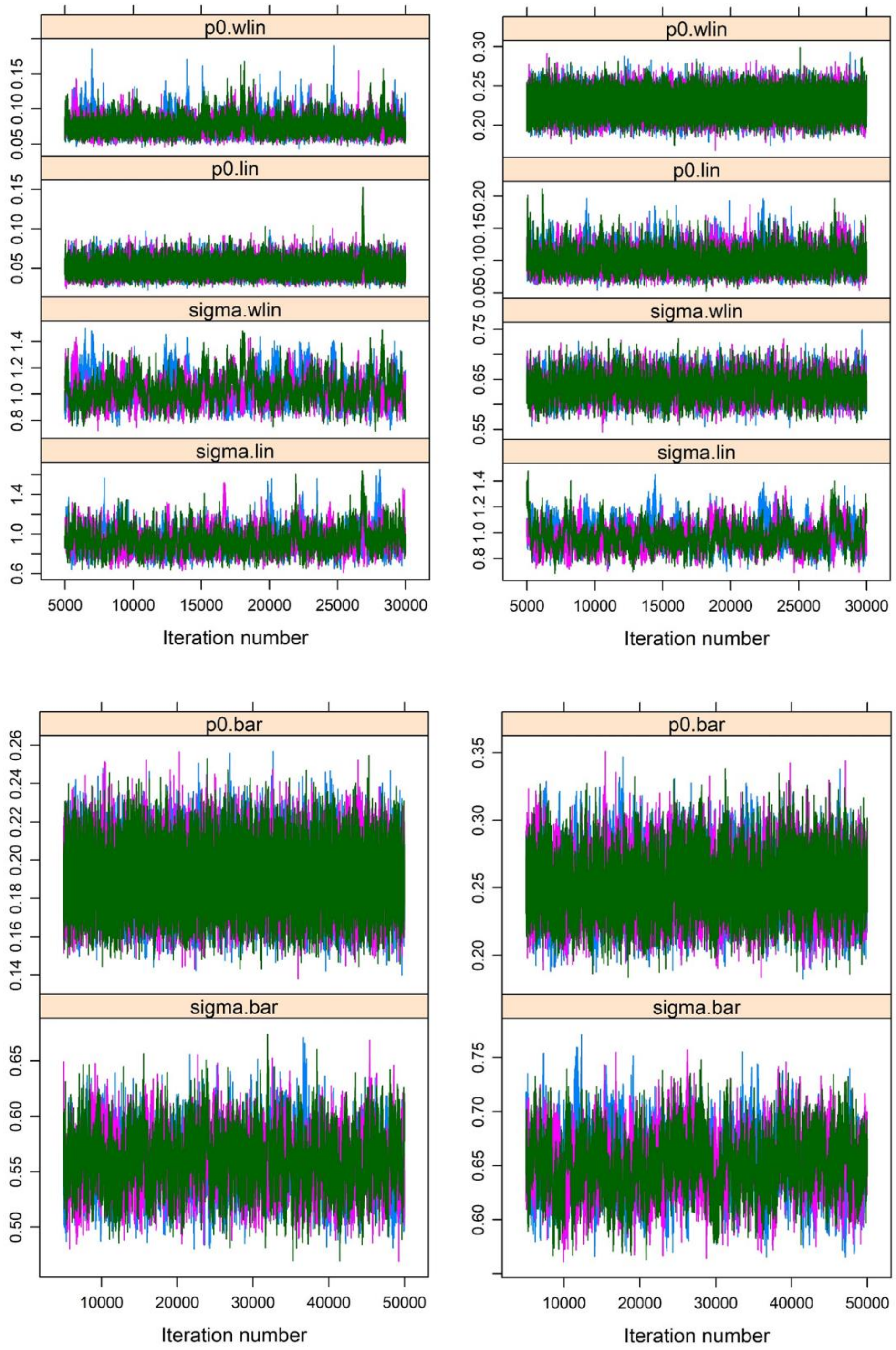

Figure S3. Convergence of Markov chains for the basal detection rate (λ_0) and movement parameters (σ) of common genet *G. genetta* (upper) and stone marten *M. foina* (lower) for the first (left) and the second study year (right) in localities with Iberian lynx presence (lin) and without lynx (wlin). For the stone marten we used a shared σ and λ_0 for all study localities.

Top-down and bottom-up effects modulate species co-existence in a context of top predator restoration

Appendix S3. Overall results from camera-trapping

Tamara Burgos[1]*, Javier Salesa[1], Jose María Fedriani[2,3], Gema Escribano-Ávila[4], José Jiménez[5], Miha Krofel[7], Inmaculada Cancio[1,6], Javier Hernández-Hernández[1,8], Javier Rodríguez-Siles[6] and Emilio Virgós[1]

[1]*Área de Biodiversidad y Conservación, Departamento de Biología, Geología, Física y Química Inorgánica, Rey Juan Carlos University, Madrid, Spain*

[2]*Centro de Investigaciones sobre Desertificación CIDE, CSIC-UVEG-GV, Carretera de Moncada a Náquera, km 4,5. 46113 Moncada (Valencia), Spain*

[3]*Estación Biológica de Doñana (EBD – CSIC), Seville, Spain*

[4]*Biodiversity, Ecology and Evolution Department. Biological Science Faculty. Universidad Complutense de Madrid. Ciudad Universitaria, C/ José Antonio Novais 12, Madrid, Spain*

[5]*Instituto de Investigación en Recursos Cinegéticos (CSIC-UCLM-JCCM), 13071 Ciudad Real, Spain*

[6]*Asociación de Estudio y Conservación de Fauna Harmusch, C/San Antón 15, 1º 13580, Almodóvar del Campo, Ciudad Real, Spain*

[7]*Department for Forestry, Biotechnical Faculty, University of Ljubljana, Ljubljana, Slovenia.*

[8]*Road Ecology Lab, Department of Biodiversity, Ecology and Evolution, Faculty of Biology, Complutense University of Madrid, Madrid, Spain*

Correspondence author: tamara.burgos@urjc.es

Table S1. Number of adult photo-identified individuals in each study locality and year.

Year	Lynx	Locality	Adult identified individuals		
			L. pardinus	*G. genetta*	*M. foina*
1	Presence	Gorgogil	3	7	0
		Puerto Bajo	3	4	1
		Valquemado	7	2	0
		Fontanarejo2	3	4	0
		Chopos	4	5	0
	Absence	Lugar Nuevo	0	8	6
		Selladores	0	10	17
		Fontanarejo1	0	6	12
		Cereceda	1	4	9
		Risquillo	0	8	5
2	Presence	Gorgogil	2	7	1
		Puerto Bajo	3	1	1
		Valquemado	6	5	0
		Fontanarejo2	2	6	2
		Chopos	2	9	2
	Absence	Lugar Nuevo	0	16	5
		Selladores	0	13	18
		Fontanarejo1	0	12	11
		Cereceda	1	4	9
		Risquillo	1	10	4

Table S2. Proportion of cameras with presence and number of independent captures by 100 cam-days of carnivores in each study locality and year.

**Year**	**Lynx**	**Locality**	**Occurrence**			**Captures/100 cam-days**		
			L. pardinus	*G. genetta*	*M. foina*	*L. pardinus*	*G. genetta*	*M. foina*
1	Presence	Gorgogil	0.50	0.42	0.00	1.82	4.19	0.00
		Puerto Bajo	0.83	0.25	0.08	3.36	1.31	0.19
		Valquemado	0.75	0.17	0.00	6.76	6.96	0.00
		Fontanarejo2	0.33	0.58	0.00	1.55	1.98	0.00
		Chopos	0.67	0.42	0.00	1.60	7.98	0.00
	Absence	Lugar Nuevo	0.00	0.92	0.67	0.00	32.24	15.61
		Selladores	0.00	1	1	0.00	10.72	50.65
		Fontanarejo1	0.00	0.83	1	0.00	12.96	54.44
		Cereceda	0.08	0.67	0.83	0.22	10.99	23.08
		Risquillo	0.00	0.58	0.67	0.00	8.43	10.73
2	Presence	Gorgogil	0.42	0.33	0.08	2.05	3.42	0.23
		Puerto Bajo	0.42	0.25	0.08	2.02	1.00	0.20
		Valquemado	0.83	0.5	0.00	5.93	9.41	0.00
		Fontanarejo2	0.42	0.50	0.08	2.13	5.23	1.16
		Chopos	0.42	0.75	0.25	1.82	21.69	0.99
	Absence	Lugar Nuevo	0.00	1	0.92	0.00	77.27	15.42
		Selladores	0.00	1	1	0.00	54.86	67.19
		Fontanarejo1	0.00	0.92	1	0.00	20.33	65.65
		Cereceda	0.08	0.42	0.83	0.23	4.28	22.52
		Risquillo	0.17	0.75	0.83	0.40	7.34	14.68

Table S3. Proportion of cameras with presence and captures by 100 cam-days of prey in each study locality and year.

Year	Lynx	Locality	Occurrence			Captures/100 cam-days		
			Rabbit	Dormouse	Other rodents	Rabbit	Dormouse	Other rodents
1	Presence	Gorgogil	0.33	0.67	0.83	8.20	19.85	39.98
		Puerto Bajo	0.58	0.83	1.00	7.46	287.13	71.46
		Valquemado	0.25	0.75	1.00	2.78	160.44	117.70
		Fontanarejo2	0.42	0.33	0.92	7.77	30.93	16.53
		Chopos	0.58	0.33	0.92	8.87	14.72	47.34
	Absence	Lugar Nuevo	0.00	0.00	0.50	0.00	0.00	4.09
		Selladores	0.00	0.00	0.42	0.00	0.00	6.28
		Fontanarejo1	0.00	0.00	0.42	0.00	0.00	4.63
		Cereceda	0.08	0.17	0.92	0.44	21.98	12.09
		Risquillo	0.08	0.17	0.92	0.19	0.96	56.71
2	Presence	Gorgogil	0.25	0.33	0.50	2.97	5.02	24.66
		Puerto Bajo	0.58	0.92	0.50	16.94	254.64	32.66
		Valquemado	0.58	0.50	0.75	7.57	20.04	22.70
		Fontanarejo2	0.33	0.75	0.92	11.24	230.62	15.50
		Chopos	0.50	0.33	0.75	2.65	1.16	17.22
	Absence	Lugar Nuevo	0.00	0.00	0.42	0.00	0.00	2.86
		Selladores	0.00	0.00	0.83	0.00	0.00	9.90
		Fontanarejo1	0.08	0.00	0.67	0.20	0.00	23.78
		Cereceda	0.00	0.00	0.50	0.00	0.00	4.50
		Risquillo	0.00	0.08	0.67	0.00	0.60	28.17

Table S4. Camera-traps working days in each study locality and year.

Lynx	Locality	Year	Date set	Working days
Presence	Gorgogil	1	19/12/2018	549
		2	14/11/2019	438
	Puerto Bajo	1	04/02/2019	536
		2	12/05/2020*	496
	Valquemado	1	18/12/2018	503
		2	10/01/2020	489
	Fontanarejo2	1	13/10/2018	708
		2	12/02/2020	516
	Chopos	1	05/12/2018	564
		2	13/11/2019	604
Absence	Lugar Nuevo	1	19/10/2018	538
		2	15/11/2019	629
	Selladores	1	17/10/2018	541
		2	20/11/2019	576
	Fontanarejo1	1	12/10/2018	540
		2	13/02/2020	492
	Cereceda	1	05/03/2019	455
		2	11/05/2020*	444
	Risquillo	1	05/02/2019	522
		2	11/02/2020	504

Table S5. Number of detections per 100 cam-days of carnivores and prey species used for temporal analysis outside and inside breeding season for carnivores. Camera-traps reached an overall effort of 4543 trap-days in sites with lynx and 3883 trap-days in sites without lynx excluding breeding season and 1024 trap-and 1415 trap-days in sites with lynx and without lynx, respectively during the breeding season.

**Species**	**Lynx**	**Detections/100 cam-days (n)**	
**Carnivores**		Non-breeding	Breeding
Iberian lynx *L. pardinus*	Absence	-	-
	Presence	2.88 (131)	2.05 (21)
Common genet *G. genetta*	Absence	31.73 (1233)	8.41 (119)
	Presence	7.26 (330)	1.76 (18)
Stone marten *M. foina*	Absence	37.24 (1446)	23.75 (336)
	Presence	0.24 (11)	0.49 (5)
**Prey**			
Rabbit *O. cuniculus*	Absence	0.05 (2)	0.14 (2)
	Presence	6.80 (309)	9.96 (102)
Garden dormouse *E. quercinus*	Absence	0.28 (11)	6.86 (97)
	Presence	73.43 (3336)	187.99 (1925)
Other rodents	Absence	14.04 (545)	15.97 (226)
	Presence	40.97 (1861)	24.22 (248)

Table S6. Proportion of visits of the common genet and the stone marten in cameras where the Iberian lynx did not appear in localities with presence of lynx.

**Locality**	***G. genetta* (Y1)**	***G. genetta* (Y2)**	***M. foina* (Y1)**	***M. foina* (Y2)**
Gorgogil	0.7391304	0.9333333	-	1
Puerto Bajo	0	0.4	0	1
Valquemado	0.6	0.6086957	-	-
Fontanarejo2	0.9285714	0.7037037	-	1
Chopos	0.1111111	0.8015267	-	1

Table S7. Average proportion of cameras where each species was exclusively active and where lynx and genet overlapped (G x L) per hour of the daily cycle. Empirical bootstrapped CI are shown in brackets.

**Species**	**Year**	**Locality**				
		Gorgogil	Puerto Bajo	Valquemado	Fontanarejo2	Chopos
G. genetta	1	0.15 (0.11-0.19)	0.08	0.14 (0.12-0.17)	0.11 (0.09-0.14)	0.14 (0.11-0.18)
G. genetta	2	0.10 (0.08-0.12)	0.10 (0.07-0.14)	0.14 (0.11-0.17)	0.14 (0.11-0.18)	0.36 (0.30-0.42)
L. pardinus	1	0.11 (0.08-0.14)	0.11 (0.09-0.14)	0.14 (0.10-0.18)	0.11 (0.08-0.14)	0.13 (0.09-0.16)
L. pardinus	2	0.08	0.11 (0.06-0.16)	0.14 (0.10-0.17)	0.11 (0.08-0.12)	0.08
G x L	1	0.08	0	0.08	0.08	0
G x L	2	0	0	0.08	0	0.08

Top-down and bottom-up effects modulate species co-existence in a context of top predator restoration

Appendix S4. R + Nimble Code for the Stone Marten (Year 1)

Tamara Burgos[1]*, Javier Salesa[1], Jose María Fedriani[2,3], Gema Escribano-Ávila[4], José Jiménez[5], Miha Krofel[7], Inmaculada Cancio[1,6], Javier Hernández-Hernández[1,8], Javier Rodríguez-Siles[6] and Emilio Virgós[1]

[1]*Área de Biodiversidad y Conservación, Departamento de Biología, Geología, Física y Química Inorgánica, Rey Juan Carlos University, Madrid, Spain*

[2]*Centro de Investigaciones sobre Desertificación CIDE, CSIC-UVEG-GV, Carretera de Moncada a Náquera, km 4,5. 46113 Moncada (Valencia), Spain*

[3]*Estación Biológica de Doñana (EBD – CSIC), Seville, Spain*

[4]*Biodiversity, Ecology and Evolution Department. Biological Science Faculty. Universidad Complutense de Madrid. Ciudad Universitaria, C/ José Antonio Novais 12, Madrid, Spain*

[5]*Instituto de Investigación en Recursos Cinegéticos (CSIC-UCLM-JCCM), 13071 Ciudad Real, Spain*

[6]*Asociación de Estudio y Conservación de Fauna Harmusch, C/San Antón 15, 1º 13580, Almodóvar del Campo, Ciudad Real, Spain*

[7]*Department for Forestry, Biotechnical Faculty, University of Ljubljana, Ljubljana, Slovenia.*

[8]*Road Ecology Lab, Department of Biodiversity, Ecology and Evolution, Faculty of Biology, Complutense University of Madrid, Madrid, Spain*

*Correspondence author: tamara.burgos@urjc.es

Define working directory

#setwd('…')

Load recquired packages

```
library(coda)
library(lattice)
library(nimble)
```

Data

```
load("AllGardunaYear1.RData")
```

Code

Define the model

```
code <- nimbleCode({

  sigma ~ dunif(0,5)    # half normal scale parameter
  p0 ~ dunif(0,1)       # baseline detection rate

  for(t in 1:T){   # Number of sites
    psi[t] ~ dunif(0,1)

    for(i in 1:M){
      # Data augmentation variables and activity centers
      z[i,t] ~ dbern(psi[t])
      S[i,1,t] ~ dunif(xlim[1,t],xlim[2,t])
      S[i,2,t] ~ dunif(ylim[1,t],ylim[2,t])
      d2[i,1:J,t] <- (S[i,1,t]-X[1:J,1,t])^2 + (S[i,2,t]-X[1:J,2,t])^2

      for(j in 1:J){   # Loop over traps
        mu[i,j,1:K,t]<-  p0*exp(-d2[i,j,t]/(2*sigma^2))*z[i,t]*oper[j,1:K
,t]

        for(k in 1:K){ # Loop over occasions
          Y4d[i,j,k,t] ~ dbern(mu[i,j,k,t])
        } # close k
      } # close j
    } # close i

  N[t]<- sum(z[1:M,t])
  D[t]<- N[t]/A[t]

  } # close t

})

str(data)
```

```
## List of 5
##  $ Y4d : num [1:150, 1:12, 1:59, 1:10] 0 0 0 0 0 0 0 0 0 0 ...
##  $ xlim: num [1:2, 1:10] -6.94 1.28 1.76 9.81 -13.47 ...
##  $ ylim: num [1:2, 1:10] -3.75 3.9 6.51 14.93 -17.19 ...
##  $ oper: num [1:12, 1:59, 1:10] 1 1 1 1 1 1 1 0 1 1 ...
##  $ X   : num [1:12, 1:2, 1:10] -3.85 -3.42 -3.94 -3.47 -3.19 ...

str(inits)

## List of 5
##  $ z    : num [1:150, 1:10] 1 1 1 1 1 1 0 0 0 0 ...
##  $ S    : num [1:150, 1:2, 1:10] 0.1138 -0.0597 0.1272 0.3372 -6.2276
...
##  $ p0   : num 0.0607
##  $ psi  : num [1:10] 0.5 0.5 0.5 0.5 0.5 0.5 0.5 0.5 0.5 0.5
##  $ sigma: num 0.478

str(constants)

## List of 5
##  $ K: num 59
##  $ M: num 150
##  $ J: int 12
##  $ T: num 10
##  $ A: num [1:10] 62.9 67.8 71.4 77.4 73.8 ...

params <- c("psi", "p0", "N", "sigma", "D")

Rmodel <- nimbleModel(code=code, constants=constants, data=data, inits=in
its, check=FALSE, calculate=FALSE)
Cmodel <- compileNimble(Rmodel)
conf<-configureMCMC(Rmodel, monitors=params, onlySlice=TRUE)

## ===== Monitors =====
## thin = 1: D, N, p0, psi, sigma
## ===== Samplers =====
## slice sampler (4512)
##   - sigma
##   - p0
##   - psi[]  (10 elements)
##   - z[]  (1500 elements)
##   - S[]  (3000 elements)

conf$removeSamplers("S")
ACnodes <- paste0("S[", 1:constants$M, ", 1:2, 1:10]")
for(node in ACnodes) {
    conf$addSampler(target = node,
                        type = "RW_block",
                        control = list(adaptScaleOnly = TRUE),
                        silent = TRUE)
}
```

```
MCMC <- buildMCMC(conf)

CompMCMC <- compileNimble(MCMC, project = Rmodel)
```

Run the model

```
nb=5000        # Burnin
ni=50000 +nb   # Iters
nc=3           # Chains


start.time2<-Sys.time()
outNim <- runMCMC(CompMCMC, niter = ni , nburnin = nb , nchains = nc, ini
ts=inits,
                  setSeed = TRUE, progressBar = TRUE, samplesAsCodaMCMC =
TRUE)

end.time<-Sys.time()
end.time-start.time
end.time-start.time2 # post-compilation run time
```

Results

```
source("summary_functions.R")
summary<-nimble_summary(outNim)  # All r-hat<1.1

## Estimates based on 3 chains of 50000 iterations

summary # to see results

##             mean     sd   2.5%    50%  97.5%  rhat
## D[1]       0.383  0.129  0.175  0.366  0.684 1.000
## D[2]       0.631  0.154  0.369  0.620  0.974 1.002
## D[3]       0.317  0.106  0.154  0.308  0.560 1.000
## D[4]       0.257  0.094  0.116  0.245  0.478 1.001
## D[5]       0.806  0.166  0.515  0.786  1.166 1.001
## D[6]       0.087  0.061  0.015  0.075  0.240 1.000
## D[7]       0.029  0.035  0.000  0.013  0.129 1.001
## D[8]       0.032  0.039  0.000  0.014  0.137 1.000
## D[9]       0.030  0.036  0.000  0.013  0.128 1.001
## D[10]      0.026  0.031  0.000  0.011  0.115 1.000
## N[1]      24.086  8.126 11.000 23.000 43.000 1.000
## N[2]      42.794 10.440 25.000 42.000 66.000 1.002
## N[3]      22.652  7.584 11.000 22.000 40.000 1.000
## N[4]      19.911  7.279  9.000 19.000 37.000 1.001
## N[5]      59.439 12.272 38.000 58.000 86.000 1.001
## N[6]       5.806  4.044  1.000  5.000 16.000 1.000
## N[7]       2.253  2.736  0.000  1.000 10.000 1.001
## N[8]       2.370  2.848  0.000  1.000 10.000 1.000
## N[9]       2.321  2.791  0.000  1.000 10.000 1.001
## N[10]      2.273  2.723  0.000  1.000 10.000 1.000
```

```
## p0.bar     0.190  0.014  0.165  0.190  0.219 1.000
## psi[1]     0.165  0.061  0.067  0.158  0.304 1.000
## psi[2]     0.288  0.078  0.155  0.282  0.458 1.002
## psi[3]     0.156  0.058  0.063  0.149  0.287 1.000
## psi[4]     0.138  0.055  0.051  0.131  0.264 1.000
## psi[5]     0.397  0.090  0.240  0.391  0.591 1.001
## psi[6]     0.045  0.031  0.005  0.038  0.124 1.000
## psi[7]     0.021  0.021  0.001  0.015  0.079 1.001
## psi[8]     0.022  0.022  0.001  0.015  0.082 1.000
## psi[9]     0.022  0.022  0.001  0.015  0.080 1.001
## psi[10]    0.022  0.021  0.001  0.015  0.079 1.000
## sigma.bar  0.557  0.024  0.514  0.556  0.605 1.004
```

Top-down and bottom-up effects modulate species co-existence in a context of top predator restoration

Appendix S5. R + Nimble Code for the Stone Marten (Year 2)

Tamara Burgos[1]*, Javier Salesa[1], Jose María Fedriani[2,3], Gema Escribano-Ávila[4], José Jiménez[5], Miha Krofel[7], Inmaculada Cancio[1,6], Javier Hernández-Hernández[1,8], Javier Rodríguez-Siles[6] and Emilio Virgós[1]

[1]Área de Biodiversidad y Conservación, Departamento de Biología, Geología, Física y Química Inorgánica, Rey Juan Carlos University, Madrid, Spain

[2]Centro de Investigaciones sobre Desertificación CIDE, CSIC-UVEG-GV, Carretera de Moncada a Náquera, km 4,5. 46113 Moncada (Valencia), Spain

[3]Estación Biológica de Doñana (EBD – CSIC), Seville, Spain

[4]Biodiversity, Ecology and Evolution Department. Biological Science Faculty. Universidad Complutense de Madrid. Ciudad Universitaria, C/ José Antonio Novais 12, Madrid, Spain

[5]Instituto de Investigación en Recursos Cinegéticos (CSIC-UCLM-JCCM), 13071 Ciudad Real, Spain

[6]Asociación de Estudio y Conservación de Fauna Harmusch, C/San Antón 15, 1º 13580, Almodóvar del Campo, Ciudad Real, Spain

[7]Department for Forestry, Biotechnical Faculty, University of Ljubljana, Ljubljana, Slovenia.

[8]Road Ecology Lab, Department of Biodiversity, Ecology and Evolution, Faculty of Biology, Complutense University of Madrid, Madrid, Spain

**Correspondence author: tamara.burgos@urjc.es*

Define working directory

#setwd('…')

Load recquired packages

```
library(coda)
library(lattice)
library(nimble)
```

Data

```
load("AllGardunaYear2.RData")
```

Code

Define the model

```
code <- nimbleCode({

  sigma ~ dunif(0,5)    # half normal scale parameter
  p0 ~ dunif(0,1)       # baseline detection rate

  for(t in 1:T){    # Number of sites
    psi[t] ~ dunif(0,1)

    for(i in 1:M){
      # Data augmentation variables and activity centers
      z[i,t] ~ dbern(psi[t])
      S[i,1,t] ~ dunif(xlim[1,t],xlim[2,t])
      S[i,2,t] ~ dunif(ylim[1,t],ylim[2,t])
      d2[i,1:J,t] <- (S[i,1,t]-X[1:J,1,t])^2 + (S[i,2,t]-X[1:J,2,t])^2

      for(j in 1:J){    # Loop over traps
        mu[i,j,1:K,t]<-  p0*exp(-d2[i,j,t]/(2*sigma^2))*z[i,t]*oper[j,1:K
,t]

        for(k in 1:K){ # Loop over occasions
          Y4d[i,j,k,t] ~ dbern(mu[i,j,k,t])
        } # close k
      } # close j
    } # close i

  N[t]<- sum(z[1:M,t])
  D[t]<- N[t]/A[t]

  } # close t

})

str(data)
```

```
## List of 5
##  $ Y4d : num [1:150, 1:12, 1:76, 1:10] 0 0 0 0 0 0 0 0 0 0 ...
##  $ xlim: num [1:2, 1:10] -6.94 1.28 1.76 9.81 -13.47 ...
##  $ ylim: num [1:2, 1:10] -3.75 3.9 6.51 14.93 -17.19 ...
##  $ oper: num [1:12, 1:76, 1:10] 1 1 1 1 1 1 1 1 1 1 ...
##  $ X   : num [1:12, 1:2, 1:10] -3.85 -3.42 -3.94 -3.47 -3.19 ...

str(inits)

## List of 5
##  $ z    : num [1:150, 1:10] 1 1 1 1 1 1 1 1 1 0 ...
##  $ S    : num [1:150, 1:2, 1:10] 1.212 -2.472 -0.152 -1.712 -3.254 ...
##  $ p0   : num 0.187
##  $ psi  : num [1:10] 0.5 0.5 0.5 0.5 0.5 0.5 0.5 0.5 0.5 0.5
##  $ sigma: num 0.539

str(constants)

## List of 5
##  $ K: num 76
##  $ M: num 150
##  $ J: int 12
##  $ T: num 10
##  $ A: num [1:10] 62.9 67.8 71.4 77.4 73.8 ...

params <- c("psi", "p0", "N", "sigma", "D")

Rmodel <- nimbleModel(code=code, constants=constants, data=data, inits=in
its, check=FALSE, calculate=FALSE)
Cmodel <- compileNimble(Rmodel)
conf<-configureMCMC(Rmodel, monitors=params, onlySlice=TRUE)

## ===== Monitors =====
## thin = 1: D, N, p0, psi, sigma
## ===== Samplers =====
## slice sampler (4512)
##   - sigma
##   - p0
##   - psi[]  (10 elements)
##   - z[]  (1500 elements)
##   - S[]  (3000 elements)

conf$removeSamplers("S")
ACnodes <- paste0("S[", 1:constants$M, ", 1:2, 1:10]")
for(node in ACnodes) {
    conf$addSampler(target = node,
                    type = "RW_block",
                    control = list(adaptScaleOnly = TRUE),
                    silent = TRUE)
}
```

```
MCMC <- buildMCMC(conf)

CompMCMC <- compileNimble(MCMC, project = Rmodel)
```

Run the model

```
nb=5000       # Burnin
ni=50000 +nb  # Iters
nc=3          # Chains


start.time2<-Sys.time()
outNim <- runMCMC(CompMCMC, niter = ni , nburnin = nb , nchains = nc, ini
ts=inits,
                  setSeed = TRUE, progressBar = TRUE, samplesAsCodaMCMC =
TRUE)

end.time<-Sys.time()
end.time-start.time
end.time-start.time2 # post-compilation run time
```

Results

```
source("summary_functions.R")
summary<-nimble_summary(outNim)  # All r-hat<1.1

## Estimates based on 3 chains of 50000 iterations

summary # to see results

##               mean    sd   2.5%    50%  97.5%  rhat
## D[1]         0.427 0.115  0.238  0.413  0.684 1.001
## D[2]         0.468 0.114  0.280  0.457  0.723 1.001
## D[3]         0.205 0.070  0.098  0.196  0.364 1.000
## D[4]         0.166 0.065  0.065  0.155  0.323 1.000
## D[5]         0.663 0.129  0.447  0.651  0.949 1.003
## D[6]         0.066 0.046  0.015  0.060  0.180 1.001
## D[7]         0.020 0.026  0.000  0.013  0.091 1.001
## D[8]         0.097 0.051  0.027  0.082  0.219 1.000
## D[9]         0.021 0.027  0.000  0.013  0.090 1.000
## D[10]        0.020 0.025  0.000  0.011  0.092 1.001
## N[1]        26.873 7.214 15.000 26.000 43.000 1.001
## N[2]        31.731 7.723 19.000 31.000 49.000 1.001
## N[3]        14.614 5.024  7.000 14.000 26.000 1.000
## N[4]        12.855 4.997  5.000 12.000 25.000 1.000
## N[5]        48.921 9.485 33.000 48.000 70.000 1.003
## N[6]         4.397 3.099  1.000  4.000 12.000 1.001
## N[7]         1.565 2.017  0.000  1.000  7.000 1.001
## N[8]         7.071 3.705  2.000  6.000 16.000 1.000
## N[9]         1.642 2.072  0.000  1.000  7.000 1.000
## N[10]        1.755 2.214  0.000  1.000  8.000 1.001
## p0.bar       0.251 0.019  0.216  0.250  0.291 1.002
## psi[1]       0.183 0.057  0.089  0.178  0.310 1.001
```

```
## psi[2]     0.215 0.061  0.113  0.210  0.348 1.001
## psi[3]     0.103 0.041  0.038  0.097  0.196 1.000
## psi[4]     0.091 0.040  0.030  0.086  0.184 1.000
## psi[5]     0.329 0.073  0.201  0.324  0.485 1.002
## psi[6]     0.036 0.025  0.004  0.030  0.100 1.001
## psi[7]     0.017 0.017  0.000  0.012  0.062 1.001
## psi[8]     0.053 0.030  0.011  0.047  0.126 1.000
## psi[9]     0.017 0.017  0.000  0.012  0.063 1.000
## psi[10]    0.018 0.018  0.000  0.013  0.067 1.001
## sigma.bar  0.651 0.025  0.602  0.651  0.703 1.002
```

Top-down and bottom-up effects modulate species co-existence in a context of top predator restoration

Appendix S6. R + Nimble Code for the Common Genet (Year 1)

Tamara Burgos[1]*, Javier Salesa[1], Jose María Fedriani[2,3], Gema Escribano-Ávila[4], José Jiménez[5], Miha Krofel[7], Inmaculada Cancio[1,6], Javier Hernández-Hernández[1,8], Javier Rodríguez-Siles[6] and Emilio Virgós[1]

[1]Área de Biodiversidad y Conservación, Departamento de Biología, Geología, Física y Química Inorgánica, Rey Juan Carlos University, Madrid, Spain

[2]Centro de Investigaciones sobre Desertificación CIDE, CSIC-UVEG-GV, Carretera de Moncada a Náquera, km 4,5. 46113 Moncada (Valencia), Spain

[3]Estación Biológica de Doñana (EBD – CSIC), Seville, Spain

[4]Biodiversity, Ecology and Evolution Department. Biological Science Faculty. Universidad Complutense de Madrid. Ciudad Universitaria, C/ José Antonio Novais 12, Madrid, Spain

[5]Instituto de Investigación en Recursos Cinegéticos (CSIC-UCLM-JCCM), 13071 Ciudad Real, Spain

[6]Asociación de Estudio y Conservación de Fauna Harmusch, C/San Antón 15, 1º 13580, Almodóvar del Campo, Ciudad Real, Spain

[7]Department for Forestry, Biotechnical Faculty, University of Ljubljana, Ljubljana, Slovenia.

[8]Road Ecology Lab, Department of Biodiversity, Ecology and Evolution, Faculty of Biology, Complutense University of Madrid, Madrid, Spain

**Correspondence author: tamara.burgos@urjc.es*

Define working directory

#setwd('…')

Load recquired packages

```
library(coda)
library(lattice)
library(nimble)
```

Data

```
load("AllGenetYear1.RData")
```

Code

Define the model

```
code <- nimbleCode({

  sigma.lin ~ dunif(0,5)    # sigma in sites with Iberian lynx
  sigma.wlin ~ dunif(0,5)   #     "    "    "   without lynx
  p0.lin ~ dunif(0,5)       # p0 in sites with Iberian lynx
  p0.wlin ~ dunif(0,5)      #  "  "    "   without lynx

  for(t in 1:5){
    sigma[t]<- sigma.wlin
    p0[t]  <- p0.wlin
  }

  for(t in 6:10){
    sigma[t]<- sigma.lin
    p0[t] <- p0.lin
  }

  for(t in 1:T){
    psi[t] ~ dunif(0,1) # data augmentation parameter

    for(i in 1:M){
      # Data augmentation variables and activity centers
      z[i,t] ~ dbern(psi[t])
      S[i,1,t] ~ dunif(xlim[1,t],xlim[2,t])
      S[i,2,t] ~ dunif(ylim[1,t],ylim[2,t])
      d2[i,1:J,t] <- (S[i,1,t]-X[1:J,1,t])^2 + (S[i,2,t]-X[1:J,2,t])^2

      for(j in 1:J){  # loop over traps
        mu[i,j,1:K,t]<-  p0[t]*exp(-d2[i,j,t]/(2*sigma[t]^2))*z[i,t]*oper
[j,1:K,t]    # Notice trap operation flag here

        for(k in 1:K){  # loop over occasions
          Y4d[i,j,k,t] ~ dbern(mu[i,j,k,t])
        } # close k
```

```
      } # close j
    } # close i

  N[t]<- sum(z[1:M,t])
  D[t]<- N[t]/A[t]

  } # close t

})

str(data)

## List of 5
##  $ Y4d : num [1:150, 1:12, 1:59, 1:10] 0 0 0 0 0 0 0 0 0 0 ...
##  $ xlim: num [1:2, 1:10] -6.94 1.28 1.76 9.81 -13.47 ...
##  $ ylim: num [1:2, 1:10] -3.75 3.9 6.51 14.93 -17.19 ...
##  $ oper: num [1:12, 1:59, 1:10] 1 1 1 1 1 1 1 0 1 1 ...
##  $ X   : num [1:12, 1:2, 1:10] -3.85 -3.42 -3.94 -3.47 -3.19 ...

str(inits)

## List of 7
##  $ z         : num [1:150, 1:10] 1 1 1 1 1 1 0 0 0 0 ...
##  $ S         : num [1:150, 1:2, 1:10] 1.0252 -3.8106 1.0935 0.4907 -0.
0845 ...
##  $ p0.lin    : num 0.0617
##  $ p0.wlin   : num 0.0864
##  $ psi       : num [1:10] 0.5 0.5 0.5 0.5 0.5 0.5 0.5 0.5 0.5 0.5
##  $ sigma.lin : num 0.498
##  $ sigma.wlin: num 0.488

str(constants)

## List of 5
##  $ K: num 59
##  $ M: num 150
##  $ J: int 12
##  $ T: num 10
##  $ A: num [1:10] 62.9 67.8 71.4 77.4 73.8 ...

params <- c("psi", "p0.lin","p0.wlin",  "N", "sigma.lin", "sigma.wlin", "
D")

Rmodel <- nimbleModel(code=code, constants=constants, data=data, inits=in
its, check=FALSE, calculate=FALSE)
Cmodel <- compileNimble(Rmodel)
conf<-configureMCMC(Rmodel, monitors=params, onlySlice=TRUE, thin=5)

## ===== Monitors =====
## thin = 5: D, N, p0.lin, p0.wlin, psi, sigma.lin, sigma.wlin
## ===== Samplers =====
```

```
## slice sampler (4514)
##   - sigma.lin
##   - sigma.wlin
##   - p0.lin
##   - p0.wlin
##   - psi[]  (10 elements)
##   - z[]  (1500 elements)
##   - S[]  (3000 elements)

conf$removeSamplers("S")
ACnodes <- paste0("S[", 1:constants$M, ", 1:2, 1:10]")
for(node in ACnodes) {
    conf$addSampler(target = node,
                    type = "RW_block",
                    control = list(adaptScaleOnly = TRUE),
                    silent = TRUE)
}

MCMC <- buildMCMC(conf)

CompMCMC <- compileNimble(MCMC, project = Rmodel)
```

Run the model

```
nb=10000       # Burnin
ni=150000 +nb  # Iters
nc=1          # Chains

set.seed(2)
start.time2<-Sys.time()

outNim <- runMCMC(CompMCMC, niter = ni , nburnin = nb , nchains = nc,
                  setSeed = FALSE, progressBar = TRUE, samplesAsCodaMCMC
= TRUE)

end.time<-Sys.time()
end.time-start.time
end.time-start.time2 # post-compilation run time
```

Results

```
source("summary_functions.R")
summary<-nimble_summary(outNim)  # All r-hat<1.1

## Estimates based on 3 chains of 30000 iterations

summary # to see results

##               mean    sd   2.5%    50%  97.5%  rhat
## D[1]         0.194 0.064  0.095  0.175  0.350 1.002
## D[2]         0.173 0.056  0.089  0.162  0.310 1.002
## D[3]         0.224 0.066  0.126  0.210  0.378 1.003
## D[4]         0.216 0.064  0.116  0.207  0.362 1.002
```

```
## D[5]         0.271 0.074  0.163  0.258  0.447 1.002
## D[6]         0.164 0.069  0.075  0.150  0.329 1.000
## D[7]         0.080 0.044  0.026  0.065  0.194 1.000
## D[8]         0.155 0.065  0.068  0.137  0.315 1.000
## D[9]         0.172 0.065  0.077  0.167  0.321 1.000
## D[10]        0.217 0.071  0.115  0.207  0.390 1.000
## N[1]        12.203 4.056  6.000 11.000 22.000 1.002
## N[2]        11.754 3.825  6.000 11.000 21.000 1.002
## N[3]        15.990 4.687  9.000 15.000 27.000 1.003
## N[4]        16.722 4.974  9.000 16.000 28.000 1.002
## N[5]        19.962 5.425 12.000 19.000 33.000 1.002
## N[6]        10.922 4.579  5.000 10.000 22.000 1.000
## N[7]         6.160 3.370  2.000  5.000 15.000 1.000
## N[8]        11.322 4.727  5.000 10.000 23.000 1.000
## N[9]        13.377 5.052  6.000 13.000 25.000 1.000
## N[10]       18.926 6.223 10.000 18.000 34.000 1.000
## p0.lin       0.050 0.009  0.035  0.049  0.070 1.001
## p0.wlin      0.076 0.012  0.058  0.074  0.104 1.010
## psi[1]       0.087 0.035  0.033  0.082  0.168 1.001
## psi[2]       0.084 0.033  0.032  0.079  0.162 1.001
## psi[3]       0.112 0.040  0.049  0.107  0.203 1.002
## psi[4]       0.117 0.042  0.051  0.111  0.212 1.002
## psi[5]       0.138 0.045  0.065  0.133  0.240 1.001
## psi[6]       0.079 0.037  0.024  0.073  0.167 1.000
## psi[7]       0.047 0.028  0.009  0.042  0.116 1.000
## psi[8]       0.081 0.038  0.025  0.075  0.171 1.000
## psi[9]       0.095 0.041  0.033  0.089  0.191 1.000
## psi[10]      0.131 0.049  0.054  0.125  0.244 1.000
## sigma.lin    0.925 0.114  0.746  0.910  1.186 1.000
## sigma.wlin   1.023 0.105  0.852  1.010  1.259 1.015
```

Top-down and bottom-up effects modulate species co-existence in a context of top predator restoration

Appendix S7. R + Nimble Code for the Common Genet (Year 2)

Tamara Burgos[1]*, Javier Salesa[1], Jose María Fedriani[2,3], Gema Escribano-Ávila[4], José Jiménez[5], Miha Krofel[7], Inmaculada Cancio[1,6], Javier Hernández-Hernández[1,8], Javier Rodríguez-Siles[6] and Emilio Virgós[1]

[1]Área de Biodiversidad y Conservación, Departamento de Biología, Geología, Física y Química Inorgánica, Rey Juan Carlos University, Madrid, Spain

[2]Centro de Investigaciones sobre Desertificación CIDE, CSIC-UVEG-GV, Carretera de Moncada a Náquera, km 4,5. 46113 Moncada (Valencia), Spain

[3]Estación Biológica de Doñana (EBD – CSIC), Seville, Spain

[4]Biodiversity, Ecology and Evolution Department. Biological Science Faculty. Universidad Complutense de Madrid. Ciudad Universitaria, C/ José Antonio Novais 12, Madrid, Spain

[5]Instituto de Investigación en Recursos Cinegéticos (CSIC-UCLM-JCCM), 13071 Ciudad Real, Spain

[6]Asociación de Estudio y Conservación de Fauna Harmusch, C/San Antón 15, 1º 13580, Almodóvar del Campo, Ciudad Real, Spain

[7]Department for Forestry, Biotechnical Faculty, University of Ljubljana, Ljubljana, Slovenia.

[8]Road Ecology Lab, Department of Biodiversity, Ecology and Evolution, Faculty of Biology, Complutense University of Madrid, Madrid, Spain

**Correspondence author: tamara.burgos@urjc.es*

Define working directory

#setwd('...')

Load recquired packages

```
library(coda)
library(lattice)
library(nimble)
```

Data

```
load("AllGenetYear2.RData")
```

Code

Define the model

```
code <- nimbleCode({

  sigma.lin ~ dunif(0,5)    # sigma in sites with Iberian lynx
  sigma.wlin ~ dunif(0,5)   #    "    "    "   without lynx
  p0.lin ~ dunif(0,5)       # p0 in sites with Iberian lynx
  p0.wlin ~ dunif(0,5)      #  "   "    "   without lynx

  for(t in 1:5){
    sigma[t]<- sigma.wlin
    p0[t]  <- p0.wlin
  }

  for(t in 6:10){
    sigma[t]<- sigma.lin
    p0[t] <- p0.lin
  }

  for(t in 1:T){
    psi[t] ~ dunif(0,1) # data augmentation parameter

    for(i in 1:M){
      # Data augmentation variables and activity centers
      z[i,t] ~ dbern(psi[t])
      S[i,1,t] ~ dunif(xlim[1,t],xlim[2,t])
      S[i,2,t] ~ dunif(ylim[1,t],ylim[2,t])
      d2[i,1:J,t] <- (S[i,1,t]-X[1:J,1,t])^2 + (S[i,2,t]-X[1:J,2,t])^2

      for(j in 1:J){  # loop over traps
        mu[i,j,1:K,t]<-  p0[t]*exp(-d2[i,j,t]/(2*sigma[t]^2))*z[i,t]*oper
[j,1:K,t]    # Notice trap operation flag here

        for(k in 1:K){  # loop over occasions
          Y4d[i,j,k,t] ~ dbern(mu[i,j,k,t])
        } # close k
```

```
      } # close j
    } # close i

  N[t]<- sum(z[1:M,t])
  D[t]<- N[t]/A[t]

  } # close t

})

str(data)

## List of 5
##  $ Y4d : num [1:150, 1:12, 1:76, 1:10] 0 0 0 0 0 0 0 0 0 0 ...
##  $ xlim: num [1:2, 1:10] -6.94 1.28 1.76 9.81 -13.47 ...
##  $ ylim: num [1:2, 1:10] -3.75 3.9 6.51 14.93 -17.19 ...
##  $ oper: num [1:12, 1:76, 1:10] 1 1 1 1 1 1 1 1 1 1 ...
##  $ X   : num [1:12, 1:2, 1:10] -3.85 -3.42 -3.94 -3.47 -3.19 ...

str(inits)

## List of 7
##  $ z         : num [1:150, 1:10] 1 1 1 1 0 0 0 0 0 0 ...
##  $ S         : num [1:150, 1:2, 1:10] -5.805 -6.914 -5.24 0.639 -0.151
...
##  $ p0.lin    : num 0.071
##  $ p0.wlin   : num 0.124
##  $ psi       : num [1:10] 0.5 0.5 0.5 0.5 0.5 0.5 0.5 0.5 0.5 0.5
##  $ sigma.lin : num 0.541
##  $ sigma.wlin: num 0.571

str(constants)

## List of 5
##  $ K: num 59
##  $ M: num 150
##  $ J: int 12
##  $ T: num 10
##  $ A: num [1:10] 62.9 67.8 71.4 77.4 73.8 ...

 params <- c("psi", "p0.lin","p0.wlin",  "N", "sigma.lin", "sigma.wlin",
"D")

Rmodel <- nimbleModel(code=code, constants=constants, data=data, inits=in
its, check=FALSE, calculate=FALSE)
Cmodel <- compileNimble(Rmodel)
conf<-configureMCMC(Rmodel, monitors=params, onlySlice=TRUE, thin=5)

## ===== Monitors =====
## thin = 5: D, N, p0.lin, p0.wlin, psi, sigma.lin, sigma.wlin
## ===== Samplers =====
```

```
## slice sampler (4514)
##   - sigma.lin
##   - sigma.wlin
##   - p0.lin
##   - p0.wlin
##   - psi[]  (10 elements)
##   - z[]  (1500 elements)
##   - S[]  (3000 elements)

conf$removeSamplers("S")
ACnodes <- paste0("S[", 1:constants$M, ", 1:2, 1:10]")
for(node in ACnodes) {
    conf$addSampler(target = node,
                    type = "RW_block",
                    control = list(adaptScaleOnly = TRUE),
                    silent = TRUE)
}

MCMC <- buildMCMC(conf)

CompMCMC <- compileNimble(MCMC, project = Rmodel)
```

Run the model

```
nb=10000       # Burnin
ni=150000 +nb  # Iters
nc=1          # Chains


set.seed(3)
start.time2<-Sys.time()
outNim <- runMCMC(CompMCMC, niter = ni , nburnin = nb , nchains = nc,
                  setSeed = FALSE, progressBar = TRUE, samplesAsCodaMCMC
= TRUE)

end.time<-Sys.time()
end.time-start.time
end.time-start.time2 # post-compilation run time
```

Results

```
source("summary_functions.R")
summary<-nimble_summary(outNim)  # All r-hat<1.1

## Estimates based on 3 chains of 30000 iterations

summary # to see results

##               mean    sd   2.5%    50%  97.5%  rhat
## D[1]         0.217 0.085  0.095  0.207  0.413 1.000
## D[2]         0.536 0.126  0.325  0.531  0.812 1.000
## D[3]         0.639 0.130  0.420  0.630  0.924 1.001
## D[4]         0.401 0.103  0.233  0.388  0.633 1.000
```

```
## D[5]        0.510 0.114  0.325  0.502  0.759 1.000
## D[6]        0.042 0.029  0.015  0.030  0.120 1.003
## D[7]        0.135 0.047  0.065  0.129  0.246 1.005
## D[8]        0.146 0.051  0.068  0.137  0.274 1.006
## D[9]        0.229 0.062  0.141  0.218  0.372 1.006
## D[10]       0.179 0.055  0.092  0.172  0.310 1.004
## N[1]       13.641 5.345  6.000 13.000 26.000 1.000
## N[2]       36.346 8.514 22.000 36.000 55.000 1.000
## N[3]       45.636 9.293 30.000 45.000 66.000 1.001
## N[4]       31.008 7.953 18.000 30.000 49.000 1.000
## N[5]       37.641 8.413 24.000 37.000 56.000 1.000
## N[6]        2.794 1.952  1.000  2.000  8.000 1.003
## N[7]       10.434 3.608  5.000 10.000 19.000 1.005
## N[8]       10.686 3.721  5.000 10.000 20.000 1.006
## N[9]       17.831 4.814 11.000 17.000 29.000 1.006
## N[10]      15.576 4.798  8.000 15.000 27.000 1.004
## p0.lin      0.099 0.015  0.074  0.097  0.134 1.005
## p0.wlin     0.226 0.014  0.200  0.226  0.254 1.000
## psi[1]      0.096 0.043  0.032  0.090  0.196 1.000
## psi[2]      0.246 0.066  0.134  0.240  0.389 1.000
## psi[3]      0.307 0.071  0.183  0.302  0.461 1.001
## psi[4]      0.211 0.062  0.107  0.205  0.347 1.000
## psi[5]      0.254 0.066  0.141  0.249  0.397 1.000
## psi[6]      0.025 0.018  0.003  0.021  0.071 1.002
## psi[7]      0.075 0.032  0.027  0.071  0.150 1.003
## psi[8]      0.077 0.033  0.027  0.072  0.153 1.003
## psi[9]      0.124 0.041  0.057  0.119  0.217 1.004
## psi[10]     0.109 0.040  0.046  0.104  0.202 1.003
## sigma.lin   0.968 0.094  0.810  0.959  1.174 1.027
## sigma.wlin  0.635 0.023  0.593  0.634  0.683 1.001
```
